# Supplementary material for: Liver regulatory mechanisms of noncoding variants at lipid and metabolic trait loci
Source: HGG Adv. 2024 Jan 30;5(2):100275. doi: 10.1016/j.xhgg.2024.100275 (PMC10881423; doi:10.1016/j.xhgg.2024.100275)
Supplement: Document S1. Figures S1–S5 and Tables S1–S3 [file mmc1.pdf]

HGGA, Volume *5*

Supplemental information

Liver regulatory mechanisms of noncoding variants at lipid and metabolic trait loci

Gautam K. Pandey, Swarooparani Vadlamudi, Kevin W. Currin, Anne H. Moxley, Jayna C. Nicholas, Jessica C. McAfee, K. Alaine Broadaway, and Karen L. Mohlke

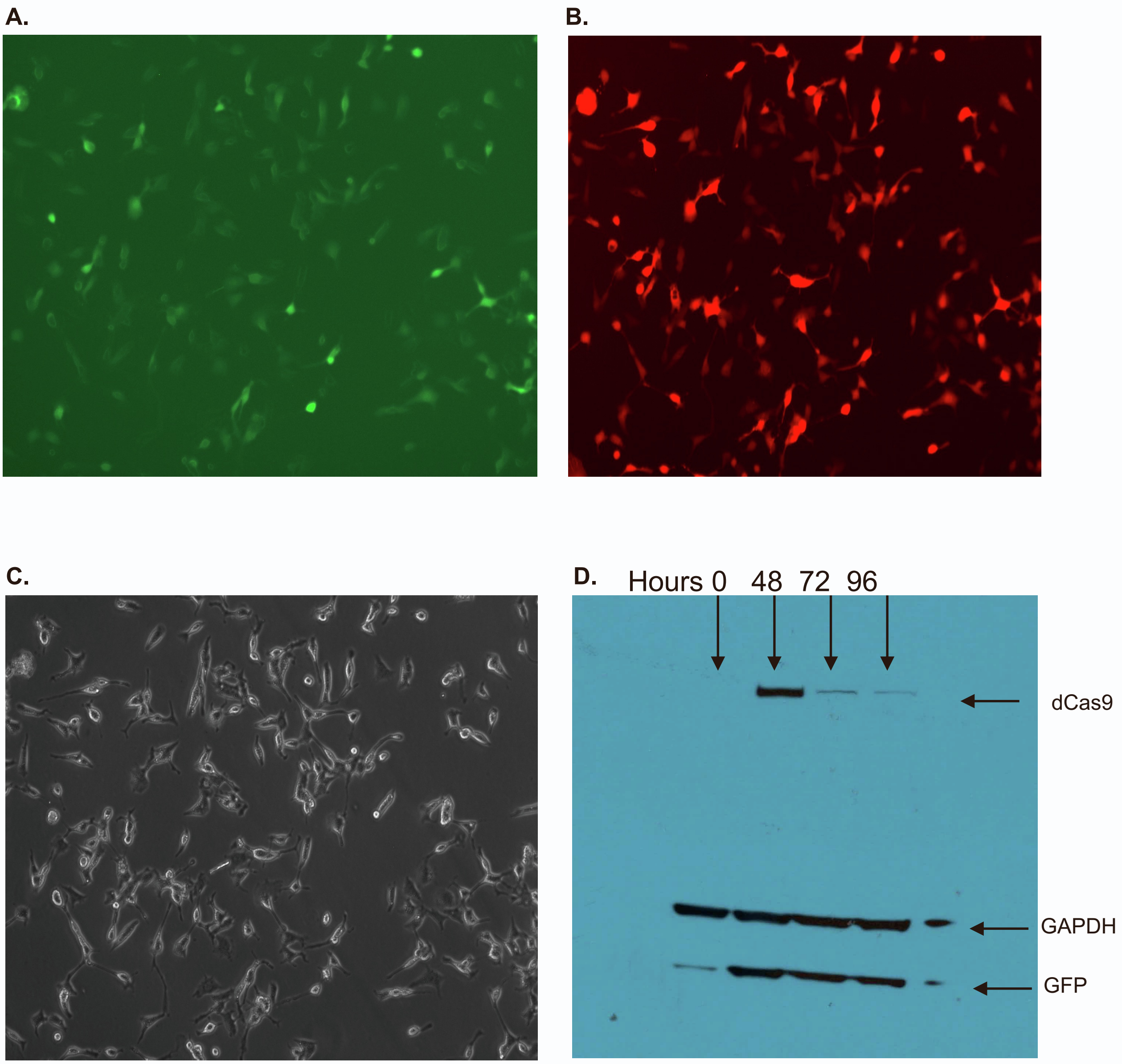

Figure S1. HepG2 cells expressing dCas9-KRAB and GFP.
(A) Expression of GFP 40 hours after activation of dCas9-KRAB and GFP by doxycycline. (B) Expression of mCherry 48 hours after infection with lentiviruses expressing sgRNAs and mCherry. (C) Bright light image of same focal area. (D) dCas9 protein levels at 48, 72 and 96 hours after activation by doxycycline. The highest expression of dCas9 protein was observed 48 hours after activation.

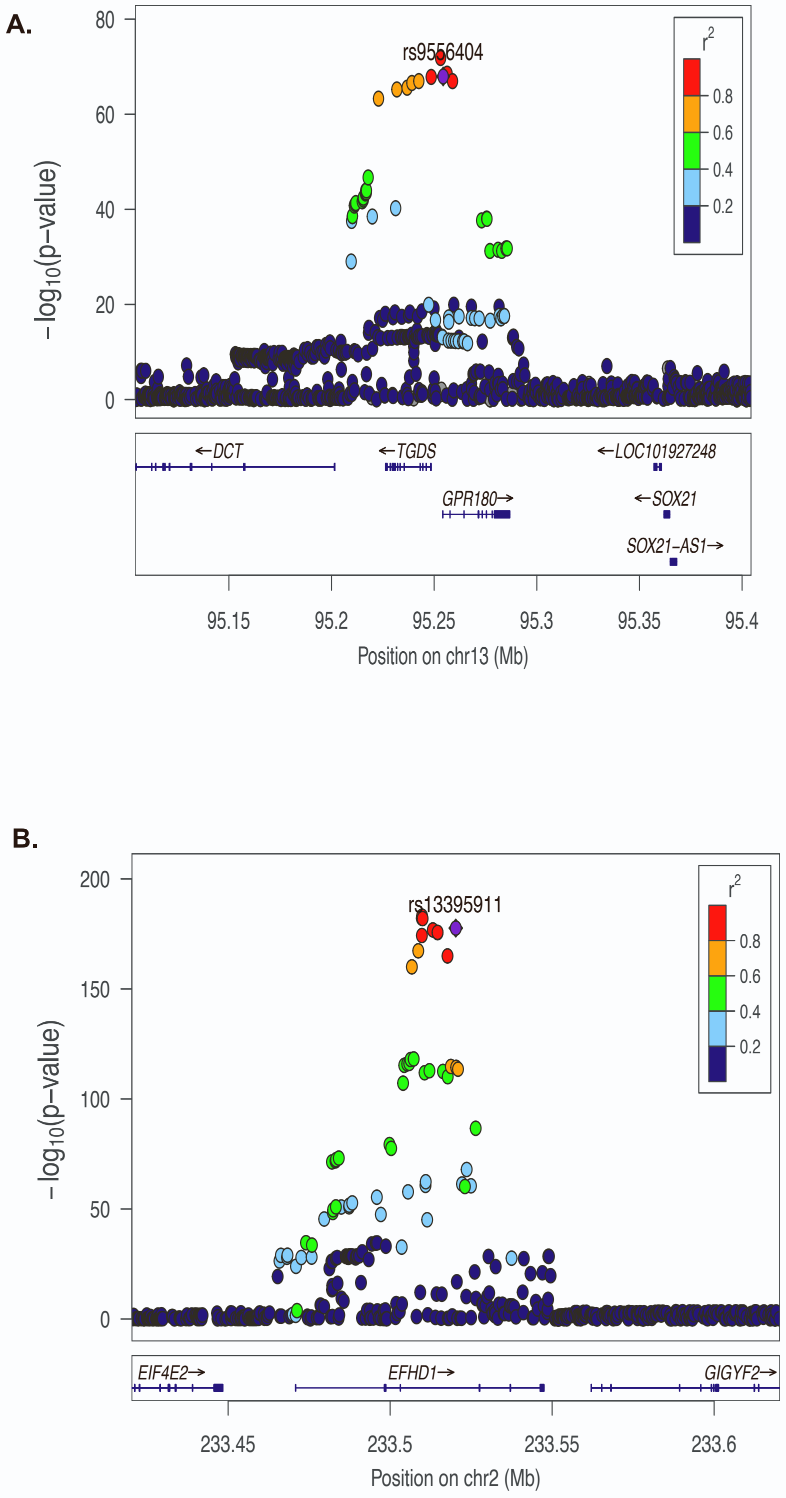

Figure S2. Liver eQTL plots for *GPR180* and *EFHD1*. Plots are colored based on LD with caQTL variants (A) rs9556404 and (B) rs13395911. Data are from Etheridge *et al.*, 2020.

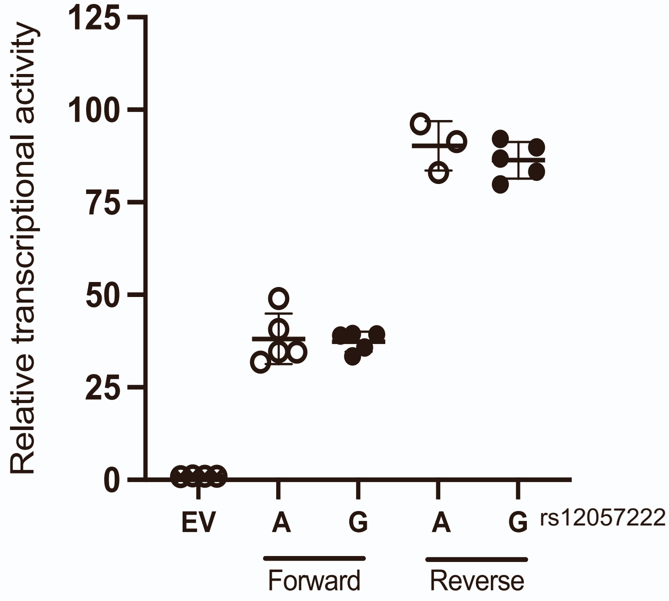

Figure S3. Transcriptional reporter activity for caQTL variant rs12057222 near *EPHA2*. A DNA element spanning rs12057222-A or rs12057222-G showed ~25 to 90-fold enhancer activity compared to empty vector (EV) control, but no allelic differences in transcriptional activity in HepG2 cells. Dots in the scatter plot represent 4-5 independent clones per allele with mean and standard deviation.

A.

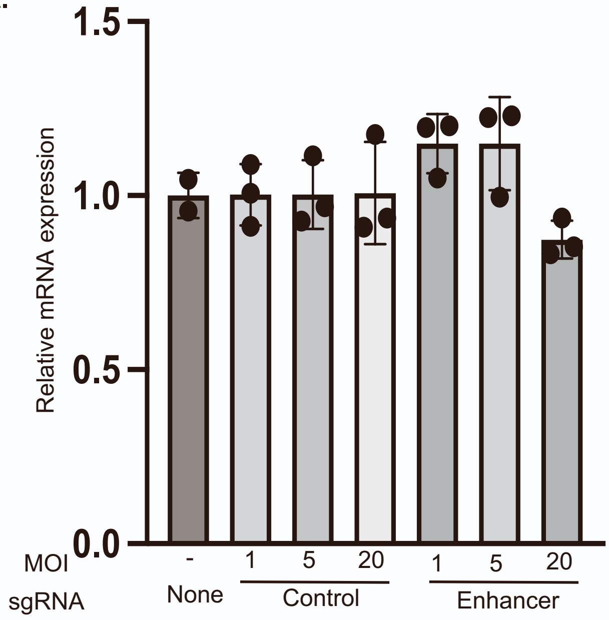

B.

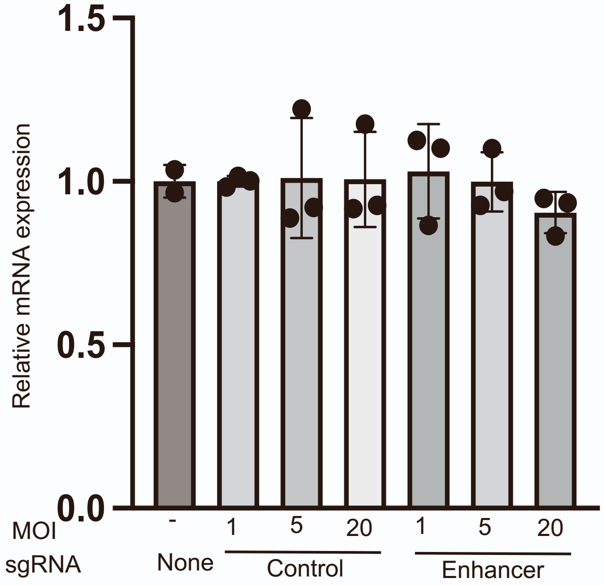

Figure S4. CRISPRi of enhancers shows less reduction of gene expression after 7 days. (A) Expression of *EFHD1* 7 days after CRISPRi of the region spanning rs13395911 at MOI of 1, 5, and 20. (B) Expression of *LITAF* 7 days after CRISPRi of the region spanning rs11644920 at MOI of 1, 5, and 20. qPCR was performed 7 days after transduction. Compared to pools of non-targeted control (Control) sgRNAs, pools of sgRNAs targeted to the enhancers did not significantly reduce gene expression. None indicates cells with no sgRNAs. Each dot represents the mean of three qPCR replicates. Bars show the mean and standard deviation of 3 biological replicates from different wells.

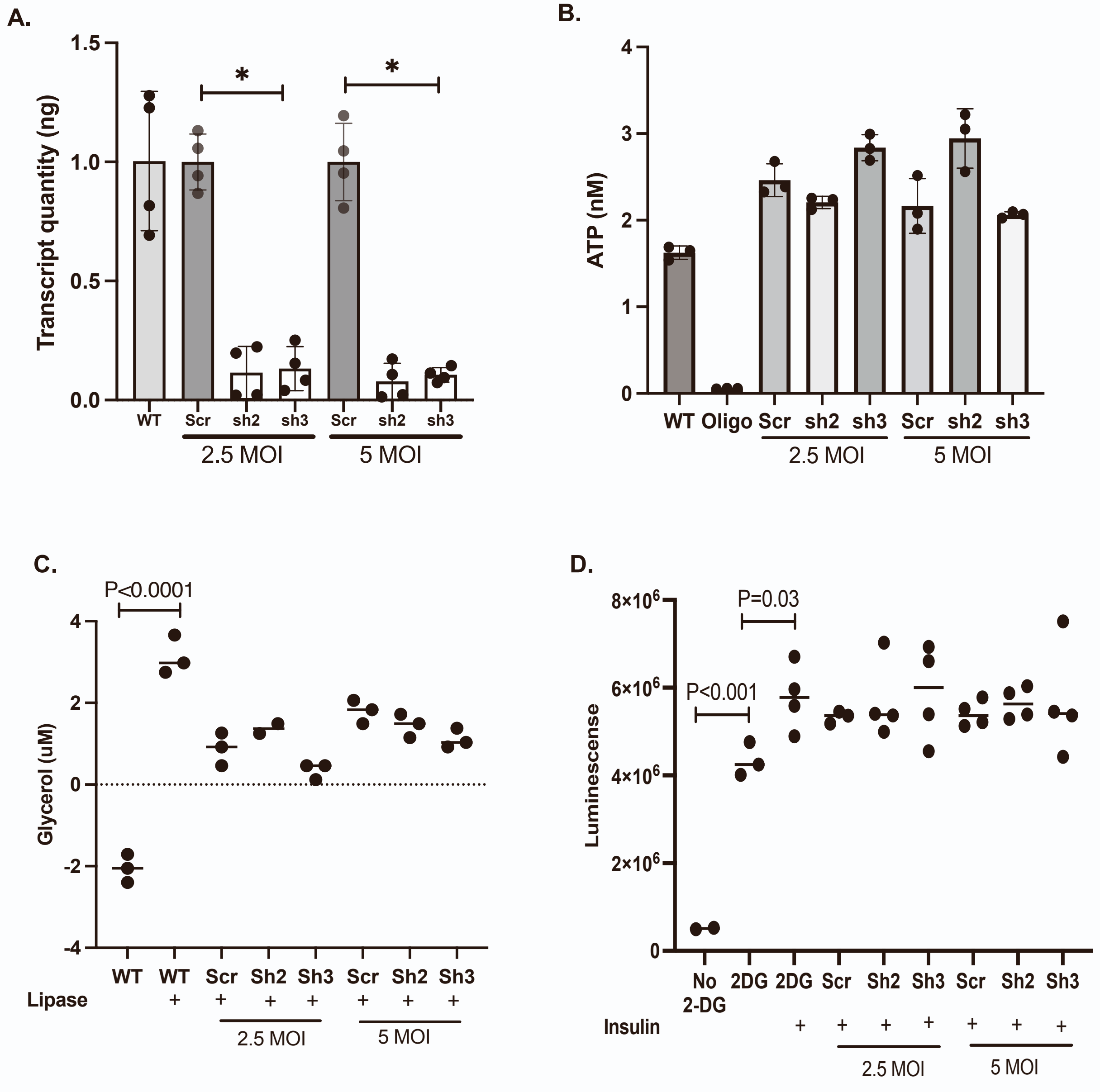

Figure S5. Effect of *EFHD1* knockdown on metabolic assays. (A) Confirmation of *EFHD1* knockdown in HepG2 cells by qPCR of *EFHD1* at 2 multiplicities of infection (MOI) with 2 shRNA constructs targeting different exons (sh2, sh3) compared to their respective scrambled controls (Scr). *P <0.05. (B) No effect of *EFHD1* knockdown on cellular ATP production. Bars represent mean and standard deviation of 3-4 biological replicates. (C) No effect on lipid accumulation after 96 hours of *EFHD1* knockdown. (D) No effect on glucose uptake after 96 hours of *EFHD1* knockdown. Scatter plot represents mean and standard deviation of 3-4 biological replicates. Each dot represents a single transduction event or biological replicate. p-values from 2-tailed t-tests.

Table S1. Characteristics of liver caQTL signals selected for study

caQTL variants studied	Lead caQTL variant	Location of caQTL variants studied	GWAS variant	Number of GWAS proxies ($r^2 \geq .8$)	GWAS p-value	Effect allele	Direction of effect on GWAS trait	References for effect on GWAS trait	eQTL gene	Direction of effect on target gene expression based on Etheridge et al 2020.	Target gene prediction
rs13395911	rs13395911	*EFHD1* intron	rs13395911	11	2E-16	T	Higher AST & ALT levels	Chen 2021, Kanai 2018	*EFHD1*	Higher *EFHD1*	eQTL, Hi-C
rs11644920	rs57792815	*LITAF* intron	rs34318965	40	3E-10	T	Higher LDL-C levels	Klarin 2018	*LITAF*	Lower *LITAF*	eQTL, Hi-C
rs34003091 & rs35081008	rs34003091	~105 bp upstream of *ZNF329*	rs34503352	6	3E-20	rs35081008-C	Higher LDL-C levels	Richardson 2020	*ZNF329*	Higher *ZNF329*	eQTL
rs9556404	rs6492720	~200 bp downstream of *GPR180* promoter	rs2298058	10	8E-09	A	Higher triglyceride levels	Hoffmann 2018	*GPR180*	Higher *GPR180*	eQTL
rs12057222	rs36086195	*EPHA2* intron	rs1497406	32	3.00E-19	G	Higher ALT levels	Chen 2021	*EPHA2*	Lower *EPHA2*	eQTL

caQTL variants studied are located within the caQTL peak, except rs12057222 is located in the adjacent peak. At ZNF329, variants rs34003091 and rs35081008 are 20 bp apart. HiC refers to promoter capture HiC (Jung et al 2019), eQTL data are from Etheridge et al., 2020. GWAS references were used to determine the direction of effect of the variant association with the GWAS traits. LDL-C, low density lipoprotein cholesterol; ALT, alanine aminotransferase; GGT, gamma glutamyltransferase; AST, aspartate aminotransferase.

Table S2. Primer sequences used in transcriptional reporter and electrophoretic mobility shift assays

Primer sequences for transcriptional reporter assays

eQTL Gene	PCR primer	Sequence (5' -- 3')	Amplified regions (hg19)
EPHA2	rs12057222_Forward<br>rs12057222_Reverse	GCGCGGTACCGGTCCTGAGTTCCCTTATCCC<br>GCGCGCTAGCCTCCCATGACCTTCCCATCC	chr1:16508335+16508728
EFHD1	rs13395911_Forward<br>rs13395911_Reverse	GCGCGGTACCGCATTGCCAAGGTACGTCTT<br>GCGCCTCGAGGCCTTCAGATGTTGCCCAG	chr2:233520112+233520454
GPR180	rs9556404_Forward<br>rs9556404_Reverse	GCGCGGTACCAGGGCAGCCAGGGTAAGAC<br>GCGCCTCGAGCACGATCACTAGTGGCGAGA	chr13:95254234+95254508
ZNF329	2SNPHap_Fwd<br>2SNPHap_Reverse	GCGCAAGCTTTCCACCCGCCAAGACTTC<br>GCGCCTCGAGTCGGGTTAAAAATCAGAGGAGA	chr19:58662049+58662332

Primers are shown 5'-3' with respect to the genome. The 2SNPHap includes rs34003091 and rs35081008

Oligonucleotide sequences for electrophoretic mobility shift assays

EFHD1	rs13395911_Forward	AACCTGTT[A/T]ACTTTGAC	chr2:233520246-233520262

Table S3. Primer sequences used to clone gRNAs

#Guide ID	Target Sequence	MIT Sperc Score	CFD Spec Score	Off Target Count	Final sequence after adding BbSI restriction site (5'--3')
EFHD1_176_Forward	TGGACACATACGGAAGGCTC *TGG*	90	94	68	CACCGTGGACACATACGGAAGGCTC
EFHD1_176_Reverse					AAACGAGCCTTCCGTATGTGTCCAC
EFHD1_141_Forward	TGTGTCCAAAACTTACAAGA *GGG*	76	87	162	CACCGTGTGTCCAAAACTTACAAG
EFHD1_141_Reverse					AAACTCTTGTAAGTTTTGGACACAC
EFHD1_240_Forward	GAGAGCTTGCACAAACCGGC *TGG*	94	96	51	CACCGAGAGCTTGCACAAACCGGC
EFHD1_240_Reverse					AAACGCCGGTTTGTGCAAGCTCTC
EFHD1_65_Forward	TCTGTTGAGAAGACGTACCT *TGG*	93	94	76	CACCGTCTGTTGAGAAGACGTACCT
EFHD1_65_Reverse					AAACAGGTACGTCTTCTCAACAGAC
EFHD1_253_Forward	TTCTCAGGTCTAGTTCCAGT *TGG*	73	83	259	CACCGTTCTCAGGTCTAGTTCCAGT
EFHD1_253_Reverse					AAACACTGGAACTAGACCTGAGAAC

#Guide ID	Target Sequence	MIT Sperc Score	CFD Spec Score	Off Target Count	Final sequence after adding BbSI restriction site (5'--3')
LITAF_128-Forward	ACTTTCATCCTGATGAGCCA *TGG*	73	86	150	CACCGACTTTCATCCTGATGAGCCA
LITAF_128-Reverse					AAACTGGCTCATCAGGATGAAAGTC
LITAF_374-Forward	AATCCACCTTTGGCACACAC *AGG*	75	91	122	CACCGAATCCACCTTTGGCACACAC
LITAF_374-Reverse					AAACGTGTGTGCCAAAGGTGGATTC
LITAF_585-Forward	TCAGCAGGAATCCTAACCTA *TGG*	82	91	91	CACCGTCAGCAGGAATCCTAACCTA
LITAF_585-Reverse					AAACTAGGTTAGGATTCCTGCTGAC
LITAF_259-Forward	GAGATCAGTAAGACGGGGGT *TGG*	84	88	139	CACCGAGATCAGTAAGACGGGGGT
LITAF_259-Reverse					AAACACCCCCGTCTTACTGATCTC
LITAF_315-Forward	ATTATTTGGGGAACTCTACT *TGG*	81	88	140	CACCGATTATTTGGGGAACTCTACT
LITAF_315-Reverse					AAACAGTAGAGTTCCCCAAATAATC
LITAF_515-Forward	TGGAAAGCTTAGGCAATAGG *AGG*	72	86	191	CACCGTGGAAAGCTTAGGCAATAGG
LITAF_515-Reverse					AAACCCTATTGCCTAAGCTTTCCAC
