# Supplementary material for: Liver regulatory mechanisms of noncoding variants at lipid and metabolic trait loci
Source: HGG Adv. 2024 Jan 30;5(2):100275. doi: 10.1016/j.xhgg.2024.100275 (PMC10881423; doi:10.1016/j.xhgg.2024.100275)
Supplement: Document S2. Article plus supplemental information [file mmc2.pdf]

HGG Advances

ARTICLE

Liver regulatory mechanisms of noncoding variants at lipid and metabolic trait loci

Gautam K. Pandey,[1] Swarooparani Vadlamudi,[1] Kevin W. Currin,[1] Anne H. Moxley,[1] Jayna C. Nicholas,[1] Jessica C. McAfee,[1,2] K. Alaine Broadaway,[1] and Karen L. Mohlke[1,3,*]

Summary

Genome-wide association studies (GWASs) have identified hundreds of risk loci for liver disease and lipid-related metabolic traits, although identifying their target genes and molecular mechanisms remains challenging. We predicted target genes at GWAS signals by integrating them with molecular quantitative trait loci for liver gene expression (eQTL) and liver chromatin accessibility QTL (caQTL). We predicted specific regulatory caQTL variants at four GWAS signals located near *EFHD1*, *LITAF*, *ZNF329*, and *GPR180*. Using transcriptional reporter assays, we determined that caQTL variants rs13395911, rs11644920, rs34003091, and rs9556404 exhibit allelic differences in regulatory activity. We also performed a protein binding assay for rs13395911 and found that FOXA2 differentially interacts with the alleles of rs13395911. For variants rs13395911 and rs11644920 in putative enhancer regulatory elements, we used CRISPRi to demonstrate that repression of the enhancers altered the expression of the predicted target and/or nearby genes. Repression of the element at rs13395911 reduced the expression of *EFHD1,* and repression of the element at rs11644920 reduced the expression of *LITAF*, *SNN*, and *TXNDC11*. Finally, we showed that *EFHD1* is a metabolically active gene in HepG2 cells. Together, these results provide key steps to connect genetic variants with cellular mechanisms and help elucidate the causes of liver disease.

Introduction

Dysregulation of lipid synthesis, metabolism, and homeostasis in the liver plays a significant role in the development of dyslipidemia, atherosclerosis, and nonalcoholic fatty liver disease, which convey a substantial healthcare burden.[1] Genome-wide association studies (GWASs) have identified hundreds of risk loci for plasma levels of liver enzymes and lipid-related metabolic traits, providing opportunities to gain insight into the pathogenesis of these diseases.[2–5] However, for the large majority of these GWAS signals, the underlying variants are mostly noncoding and lack functional data to explain how variants affect genes and how gene dysfunction leads to disease.[6] Most GWAS signals are enriched in transcriptional regulatory elements[7] and may affect chromatin accessibility, transcription factor binding, and/or gene expression.[8] Transcriptional reporter and nuclear protein binding assays can provide experimental evidence in support of allelic effects on regulatory activity.

Several bioinformatics-based analytical tools can predict candidate target genes for GWAS signals. One approach is to integrate GWAS signals with quantitative trait loci for gene expression (eQTLs) in trait-relevant tissues through colocalization analysis. Evidence for a potential regulatory mechanism is further bolstered if the colocalized GWAS and eQTL signals also colocalize with a chromatin accessibility QTL (caQTL) signal in the same tissue.[8–10] We previously integrated lipid-related metabolic trait GWAS signals with eQTL and caQTL in the liver to predict candidate variants, regulatory elements, and genes for lipid-related disorders.[11] Experimentally testing putative effector genes is particularly difficult for GWAS signals that reside in regions distal to any promoter. For these signals, CRISPR interference (CRISPRi) can be used to functionally assess potential target genes. CRISPRi has been used traditionally to repress a gene by interfering with regulatory activity by the promoter.[12,13] More recently, it has been used to repress enhancer elements to identify links between enhancers and genes.[14–16]

Here, we aimed to identify regulatory mechanisms underlying lipid-related metabolic traits in hepatocytes at five GWAS signals. We identified genetic signals that were associated with plasma lipid or liver enzyme levels and that plausibly affected gene expression regulation as identified through colocalization with liver caQTL and eQTL signals.[11,17] For selected caQTL variants, we evaluated allelic effects on regulatory activity by performing transcriptional assays. Next, we used CRISPRi to repress putative distal regulatory elements to link them with their target gene(s). Finally, we evaluated one gene, *EFHD1*, for its role in regulating metabolic activity in hepatocytes. Together, these experiments validate predicted regulatory mechanisms and provide a scalable strategy for future studies.

[1]Department of Genetics, University of North Carolina, Chapel Hill, NC 27599, USA; [2]UNC Neuroscience Center, University of North Carolina at Chapel Hill, Chapel Hill, NC 27599, USA
[3]Lead contact
*Correspondence: mohlke@med.unc.edu
https://doi.org/10.1016/j.xhgg.2024.100275.
© 2024 The Authors. This is an open access article under the CC BY license (http://creativecommons.org/licenses/by/4.0/).

Material and methods

Prediction of candidate caQTL variants and genes

As described previously,[11] we identified liver caQTL that colocalized with at least one GWAS signal for total cholesterol, triglycerides, high-density lipoprotein-cholesterol, low-density lipoprotein-cholesterol (LDL-C), or the liver enzymes ALT, aspartate aminotransferase (AST), or γ-glutamyl transferase (GGT)[5,18,19] (Table S1). We also identified candidate target genes for caQTL using proximity to promoter, colocalization with liver eQTL signals, and liver Hi-C data.[11,17,20] Here, we used ENCODE histone chromatin immunoprecipitation sequencing (ChIP-seq) data for the epigenomic marks H3K4me3 and H3K27ac to identify active promoters (H3K4me3 + H3K27ac) and enhancers (H3K27ac and distal to the transcription start sites),[21] obtained HepG2 chromatin states as ChromHMM from ENCODE,[22] and predicted transcription factors that bind at specific sites using liver ChIP-seq data from ENCODE 3.[23] We defined linkage disequilibrium (LD) between caQTL, GWAS, and eQTL variants based on 1000 Genomes European individuals.[24]

Cell culture

We obtained the HepG2 human hepatoblastoma cell line from the American Type Culture Collection (ATCC) and cultured cells in MEM-alpha (Gibco no. 12561) supplemented with 10% fetal bovine serum (FBS) and 1 mM sodium pyruvate. We obtained SNU-761 cells from the Korean Cell Line Bank and cultured them in RPMI 1640 media (Corning no. 10-040-CV) with 10% FBS. HEK293T cells from ATCC were cultured in DMEM (Sigma no. D6429) supplemented with 10% FBS and 200 mM glutamine. All of the cells were maintained at 37°C and 5% CO_2. For metabolic switching experiments, HepG2 cells were grown in DMEM (Gibco no. A14430) and SNU-761 cells were grown in RPMI 1640 (Corning) without glucose and then supplemented with 5.5 or 11.1 mM glucose or galactose for 6 days before analyzing *EFHD1* expression.

Transcriptional reporter assays

To test variants for allelic differences in transcriptional activity, we designed primers (Table S2) to span each caQTL variant and the surrounding accessible chromatin region, amplified products from human DNA, and cloned them into a firefly luciferase vector as described previously.[25,26] We cloned putative enhancer sequences into pGL4.23 (Promega) in forward and reverse orientations with respect to the genome and we cloned putative promoter elements into promoter-less vector pGL4.10 in the orientation that matches the direction of gene transcription (Table S2). We cloned and analyzed four to five sequence-verified clones for each allele. We cotransfected HepG2 cells with a luciferase reporter and phRL-TK Renilla reporter vector (Promega) using Lipofectamine 3000 (Life Technologies) following the manufacturer's protocol and measured luciferase activity 48 h after transfection using the Dual-Luciferase Reporter Assay System (Promega). We normalized luciferase to Renilla activity, calculated fold change in luciferase activity relative to an empty vector (EV; pGL4.23 or pGL4.10), and tested for differences in activity using two-tailed Student's t tests.

Electrophoretic mobility shift assays (EMSAs)

We designed and annealed biotin-labeled and unlabeled 17-bp complementary oligonucleotides centered on rs13395911 (Table S2). We conducted EMSAs using the Light Shift Chemiluminescent EMSA kit (Thermo Fisher Scientific) following the manufacturer's protocol. The binding reactions consisted of 6 μg HepG2 nuclear extract (NE-PER Kit, Thermo Fisher Scientific), 20 ng/μL poly(dI-dC), 1× binding buffer, and 400 fmol biotinylated oligonucleotides. To test the specificity of the protein complexes to each allele, we added 15-fold excess competitive unlabeled probes (Table S2) in the binding reactions. For the supershift assays, we preincubated with 4 μg of antibodies to FOXA2 (Santa Cruz, no. sc6554x) or SP1 (Santa Cruz, no. sc-17824x) in the binding reactions before performing EMSAs, as described previously.[25]

Preparation of dCas9-KRAB-NeoR stable cell line

To deploy the doxycycline-inducible CRISPRi system in HepG2 cells, we stably expressed dCas9 fused to a KRAB-repressor domain in the *AAVS1* safe-harbor locus.[27] Briefly, we electroporated 1.5 million HepG2 cells with 7.5 μg of pT077, a TRE3G-KRAB-dCas9-NeoR construct (a gift from Lindy Barret, Addgene no. 137879) and 3.75 μg of each AAVS1-TALEN-L and AAVS1-TALEN-R (gifts from Danwei Huangfu, Addgene nos. 59025 and 59026) at 1400 V, 40 ms, 2 pulses, and incubated for 15 min before transferring to a 10-cm dish in antibiotic-free media following the manufacturer's instructions (Neon Transfection System, Invitrogen). Cells were allowed to recover for 48–-72 h in regular growth media before starting selection with neomycin at 550 μg/mL for 10 days, followed by maintenance at 275 μg/mL neomycin. For experiments, we used a mixed population of cells in which ~90% of the cells expressed dCas9-KRAB based on the visualization of GFP using fluorescence microscopy. After 48–96 h of doxycycline treatment (2 μg/mL), we validated inducible dCas9 and eGFP protein expression via western blot (Figure S1). Briefly, at selected time points following doxycycline treatment, we lysed cells (150 mM NaCl, 1% NP-40, 0.5% sodium deoxycholate, 0.1% SDS, 50 mM Tris-HCl), added protease inhibitor (100×, Thermo Fisher Scientific) electrophoresed 40 μg/lane on a 4%–12% bis-acrylamide gel (Invitrogen), and transferred to polyvinylidene difluoride membrane (Millipore). We detected proteins using anti-mouse dCas9 (Millipore, no. MABE1669), anti-mouse GFP (Santa Cruz, no. sc-9996), and anti-mouse glyceraldehyde 3-phosphate dehydrogenase (Santa Cruz, no. sc-32233) at 1:750, 1:3,000, and 1:7,500 dilutions, respectively, followed by anti-mouse immunoglobulin G horseradish peroxidase (Santa Cruz, no. sc-516102) at a 1:3,000 dilution.

Selection and cloning of single-guide RNA (sgRNA) lentiviruses

We designed 12 sgRNAs (6 forward and 6 reverse) to span each regulatory element using CRISPOR (http://crispor.tefor.net/) (Table S3). We used nontargeted control (NTC) sgRNAs as described previously.[28] We cloned sgRNAs individually into the Lenti U6-sgRNA/EF1a-mCherry vector (a gift from Jeremy Day, Addgene no. 114199) using BbsI-HF and a modified cloning protocol.[29] Briefly, 30 ng of BbsSI-HF-digested and calf intestinal phosphatase-treated vector was ligated with 0.1 μM annealed and phosphorylated sgRNA oligos using T4-DNA ligase (NEB) for 4 h at 16°C. We used 2 μL of the ligated product to transform 25 μL of Stbl3 chemically competent cells (One Shot, Invitrogen) and confirmed the clones by Sanger sequencing.

Table 1. Liver caQTL variants selected for mechanistic studies

caQTL variant(s)	Variant location	GWAS variant	GWAS proxies ($r^2 \geq .8$)	caQTL- GWAS LD (r^2)	GWAS traits
rs34003091, rs35081008	*ZNF329* promoter	rs34503352	6	0.98	LDL-C
rs9556404	*GPR180* promoter	rs2298058	10	0.9	Triglycerides
rs13395911	*EFHD1* intron	rs13395911	11	1	ALT, GGT, AST
rs11644920	*LITAF* intron	rs34318965	40	0.81	LDL-C, GGT
rs12057222	*EPHA2* intron	rs1497406	32	0.82	ALT

caQTL variants indicate the candidate variants selected for study. GWAS variants indicate lead variants from GWAS signals that colocalized with the liver caQTL signals. GWAS proxies and the LD between lead caQTL and GWAS variants are based on 1000 Genomes Europeans.

We produced and functionally titered sgRNA lentiviruses as described.[30] Briefly, we grew HEK293T cells to 70% confluency and transfected them with 9.5 μg Lenti U6-sgRNA/EF1a-mCherry construct, 8 μg of packaging plasmid (psPAX2, a gift from Didier Trono, Addgene plasmid no. 12260), and 2.5 μg of an envelope plasmid (pMD2.G, a gift from Didier Trono, Addgene plasmid no. 12259) using Lipofectamine 2000 and PLUS reagent (Invitrogen) and replaced the growth media after 18 h. We harvested viral supernatants at 48 and 72 h after transfection, concentrated using Lenti-X concentrators (Clontech), titered using the Lenti-X provirus quantitation kit (Takara Bio), and represented functional titers as MOI.

CRISPRi

After plating 120,000 HepG2 cells/well onto collagen-I (Corning)-coated 12-well plates, we transduced with lenti-sgRNAs at 20 MOI, a concentration that was selected based on transduction efficiency and cell health, in the presence of 10 μg/mL polybrene media.[31,32] After 8 h, we replaced growth media with media containing doxycycline (2 μg/mL) and visualized GFP and mCherry expression by fluorescent microscopy. At 72 h or 7 days after transduction, we lysed cells, extracted total RNA (RNeasy Plus, Qiagen), and converted to cDNA (Superscript IV Vilo, Invitrogen). We assessed gene expression by qPCR using TaqMan probes (Thermo Fisher Scientific), using the $2^{-\Delta\Delta Ct}$ method and normalizing to the *B2M* expression level.

EFHD1 knockdown and cell-based functional assays

We performed short hairpin RNA (shRNA)-based lentiviral-mediated gene knockdown of *EFHD1* in HepG2 cells using four shRNA lentiviral plasmid constructs (TL304828, Origene Technologies). We used this system to test gene function because shRNA-based lentiviruses show stronger and more stable knockdown of genes.[33] Lentivirus particles expressing shRNAs were prepared and titrated as described above. At 72 h after transduction, we assessed knockdown efficiency normalized to *ACTB* by qPCR and selected the two most efficient shRNAs for functional assays. Briefly, we transduced cells for 72 h at 2.5 and 5 MOI before replating them for functional assays. We performed ATP assays (Roche HS II) in 4-h serum-starved cells in media supplemented with 10 mM galactose[34] at a density of 15,000 cells/well of 96-well plates, and we assayed lipid accumulation (Promega) at a density of 80,000 cells/well in a 96-well plate. After starving cells of serum overnight, we measured glucose uptake in cells at a density of 60,000 cells/well in a 24-well plate; we then starved the cells of glucose for 1.5 h, preincubated with human insulin (Sigma) in Krebs Ringer Buffer[35] for 30 min,[36,37] and then we measured glucose uptake (Promega).

Results

Selection of candidate regulatory variants at GWAS signals

Our previous study identified liver caQTL signals that colocalized with GWAS signals for lipid or liver metabolism and with liver eQTL signals.[11] Because caQTL variants could be located up to 1 kb from the region of chromatin accessibility,[11] we focused on variants located within the accessible region and the genes identified by the colocalized eQTL.[17] We prioritized five signals for functional studies based on the expression level of the eQTL gene in hepatocytes, H3K27ac and H3K4me3 histone modifications overlapping the accessible chromatin region,[21,38,39] 40 or fewer LD proxy variants, and prior evidence that the gene may act in lipid metabolism or liver function.[40–43] The signals were associated with LDL-C, triglycerides, ALT, GGT, and/or AST levels,[5,18,44] and the genes for the colocalized eQTL signals are *ZNF329*, *GPR180*, *EFHD1*, *LITAF*, and *EPHA2* (Figure S2; Tables 1 and S1).

The signals correspond to genes that may influence metabolism. One GWAS signal for LDL-C colocalized with a liver caQTL signal and a liver eQTL for *ZNF329* (Figures 1A–1C),[17,19] and the lead caQTL variant rs34003091 and proxy variant rs35081008 overlap a weak promoter chromatin state in HepG2 (Figure 1D) and liver ChIP-seq regions for 7 transcription factors (Figure 1E). *ZNF329* is significantly downregulated in skeletal muscle after weight loss surgery in obese humans.[45] A GWAS signal for triglycerides colocalized with an eQTL for *GPR180*, which has been reported to play a role in vascular modeling[46] and is required for the proper functioning of brown adipocytes.[43] A GWAS signal for AST and ALT colocalized with an eQTL for *EFHD1*, which codes for a mitochondrial protein known to regulate metabolic activity in B cells and neuronal cells[41,42] A GWAS signal for LDL-C[5] (Table S1) colocalized with an eQTL for *LITAF*, which is a BCL6 target gene that has been shown to enhance autophagy in B cell lymphomas.[47] At this signal, we previously demonstrated that rs11644920, which is an LD proxy ($r^2 = 0.98$) of the lead caQTL variant rs57792815, exhibited significant allelic differences in transcriptional activity and allele-specific protein binding.[11] Finally, a GWAS signal for ALT colocalized with an eQTL for

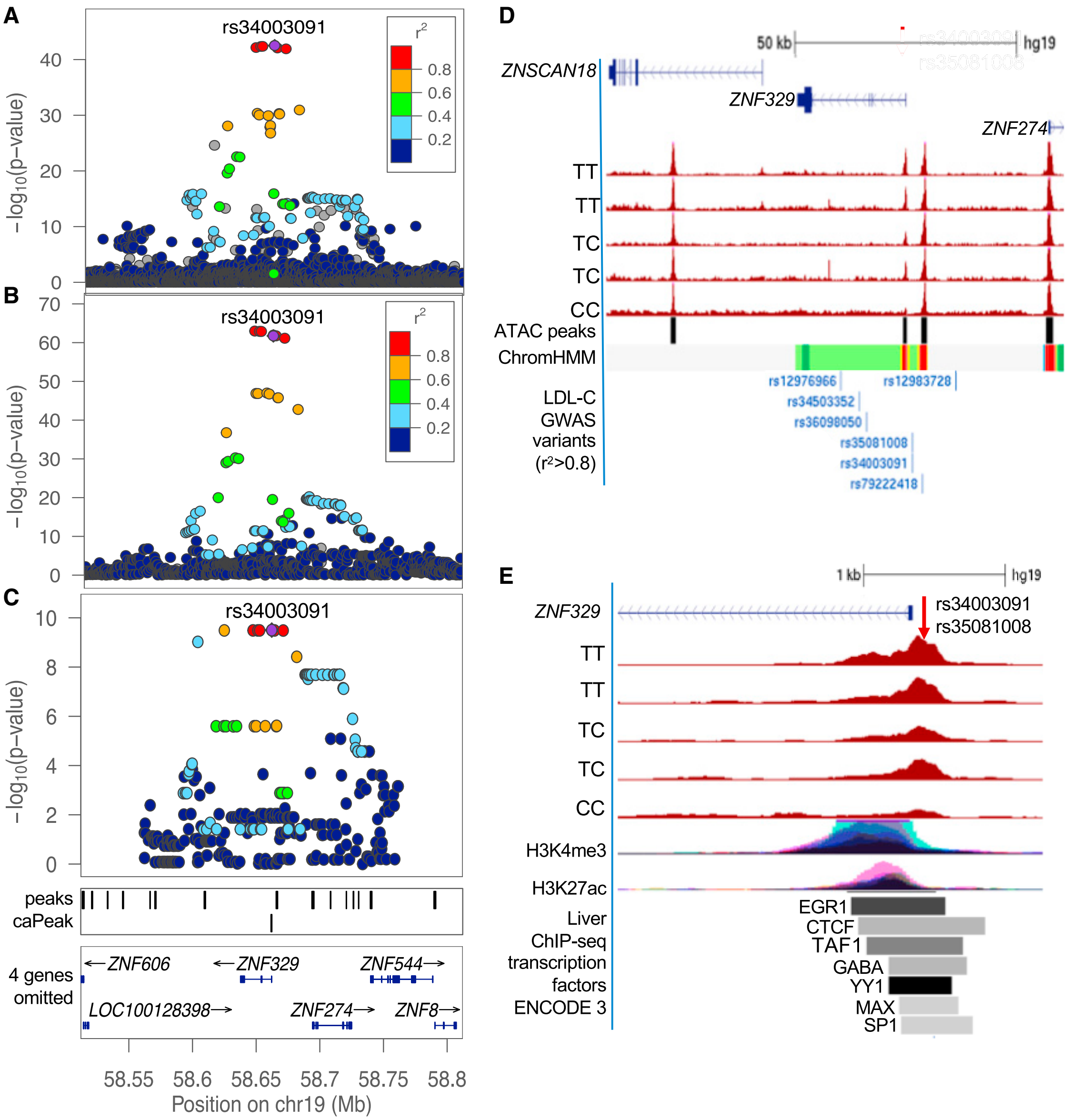

Figure 1. A plausible regulatory mechanism at a locus for LDL-C levels

(A–C) rs34003091 association (A) with plasma levels of LDL-C in the multipopulation analysis from Graham et al.,[3] (B) liver eQTL for *ZNF329* from Etheridge et al.,[17] and (C) caQTL associations from Currin et al.[11] The colors are based on the LD (r^2) with rs34003091 (purple diamonds) in 1000 Genomes Europeans. The x axis shows the GRCh37/hg19 genomic position in Mb and protein-coding genes, and the y axis shows variant-trait association in $-\log_{10}$(p value).

(D) rs34003091 and rs35081008 (20 bp apart, red arrow) overlap a liver caQTL peak at the *ZNF329* promoter and a promoter chromatin state of HepG2 cells. The region shown includes all LDL-C GWAS variants in strong LD ($r^2 > 0.8$) with rs34003091.

(E) rs34003091 and rs35081008 overlap liver ChIP-seq regions for transcription factors and chromatin marks of active enhancers.

EPHA2, which encodes a tyrosine kinase receptor implicated in several clinical and biological processes.[48,49] At this signal, rs12057222, which is an LD proxy ($r^2 = 0.82$) of the lead caQTL variant rs36086195, is not located in the chromatin accessibility region of the caQTL, but in a nearby accessible chromatin region.

Effects of caQTL variant alleles on transcriptional activity

To evaluate the putative functional caQTL variants for allelic effects on transcriptional activity, we performed transcriptional reporter assays in HepG2 cells. We tested regions around the caQTL lead signals near *ZNF329*, *GPR180*, *EFHD1*, and *EPHA2* (Table S2). All four elements showed 2- to 150-fold higher transcriptional activity than an EV control, demonstrating that the elements are promoters or enhancers and not repressors (Figures 2 and S3). The elements near *GPR180* (p = 0.008, forward; p = 0.016, reverse) *ZNF329* (p = 0.02 reverse), and *EFHD1* (p < 0.001 forward; p < 0.001 reverse) showed significant allelic differences in transcriptional activity (Figure 2). Most dramatically, at *EFHD1*, a 343-bp genomic segment containing the rs13395911-T allele showed a 3-fold

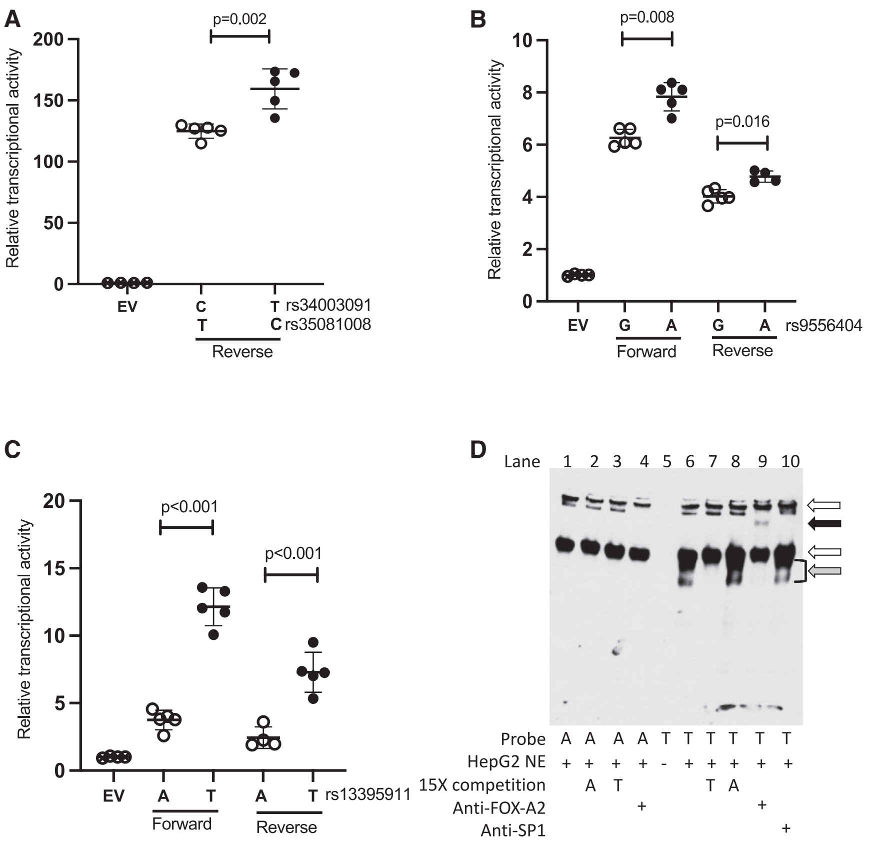

Figure 2. Allelic differences in transcriptional function for variants at 3 loci
(A–C) Transcriptional reporter activity in HepG2 cells. Transcriptional activity is normalized to empty vector (EV). Dots represent 4-5 independent clones, error bars indicate standard deviations, and p-values are from 2-tailed t-tests. (A) A 284-bp element spanning rs34003091-T and rs35081008-C near *ZNF329* showed increased transcriptional activity compared to rs34003091-C and rs35081008-T.
(B) A 331-bp element spanning rs9556404-A near *GPR180* showed increased transcriptional activity compared to rs9556404-G in both forward and reverse orientations with respect to the genome.
(C) A 343-bp element spanning rs13395911-T near *EFHD1* showed stronger transcriptional activity compared to rs13395911-A in both orientations.
(D) EMSA using HepG2 nuclear lysate and a probe spanning rs13395911 near *EFHD1* showed evidence of differential allelic protein binding (gray arrow). Addition of antibodies to FOXA2 showed a supershift (black arrow, lane 9). The white arrows indicate nonallele-specific protein binding.

increase in transcriptional activity compared to the rs13395911-A allele ($p < 0.001$). For all three signals, the alleles that showed more enhancer activity were the alleles associated with greater chromatin accessibility[11] and higher gene expression in the liver eQTL data[17] (Table S1). The element at *EPHA2* showed relatively modest transcriptional activity and no allelic differences (Figure S3); we did not pursue this element for additional analyses.

Differential binding of nuclear proteins to rs13395911 alleles

Due to the strong allelic differences observed in the transcriptional activity of the *EFHD1* element containing rs13395911, we further investigated whether transcription factors may bind differentially to the rs13395911 alleles. We performed EMSAs using HepG2 nuclear protein extract and 17-bp biotinylated probes centered around either rs13395911-A or rs13395911-T (Table S2). The rs13395911-T allele probe showed a band with stronger protein binding than the rs13395911-A allele probe (Figure 2D, gray arrow; lane 6 vs. lane 1). The addition of the excess unlabeled rs13395911-T probe competed away the band better than the excess unlabeled rs13395911-A probe (Figure 2D, lane 7 vs. lane 8), providing further evidence that the protein binds preferentially to the rs13395911-T allele. Because the genomic sequence surrounding rs13395911 has been shown to overlap transcription factor ChIP-seq data[21,23,50] for FOXA2 and SP1, we tested whether the band could correspond to one of these factors. Incubation of the DNA-protein complexes with antibodies to FOXA2 showed a supershift of the T-allele complex (black arrow; lane 9), providing evidence that FOXA2 can bind to rs13395911-T; antibodies to SP1 showed no supershift. These results suggest that FOXA2, a transcription factor known to play a role in liver development and function,[51] may mediate the differential allelic effects of rs13395911 on transcriptional activity in hepatocytes.

Regulatory element links to genes

For two signals, the caQTL variants are not located at promoters. To validate candidate effector genes for the caQTL elements spanning rs13395911 in an *EFHD1* intron and rs11644920 in an *LITAF* intron, we used CRISPRi with lenti-sgRNAs in HepG2 cells expressing dCas9-KRAB (Figure S1; Table S3). We repressed the intronic elements spanning rs13395911 and rs11644920, and after 72 h, we measured their effects on the expression level of *EFHD1*, *LITAF*, and two of the next closest genes (Figures 3 and 4). We also measured the expression level of these genes 7 days after transduction, but repression efficiency was poor (Figure S4), suggesting that the growth of unmodified cells over 7 days moderated the effect of repression. At *EFHD1*, compared to control cells transduced with NTC sgRNAs, cells with sgRNAs targeting the putative enhancer spanning rs13395911 showed 24% lower *EFHD1* expression ($p = 0.006$) (Figure 3). Two additional genes residing within 50 kb of rs13395911, *EIF4E2*, and *GIGFY* showed no change in expression ($p > 0.05$)

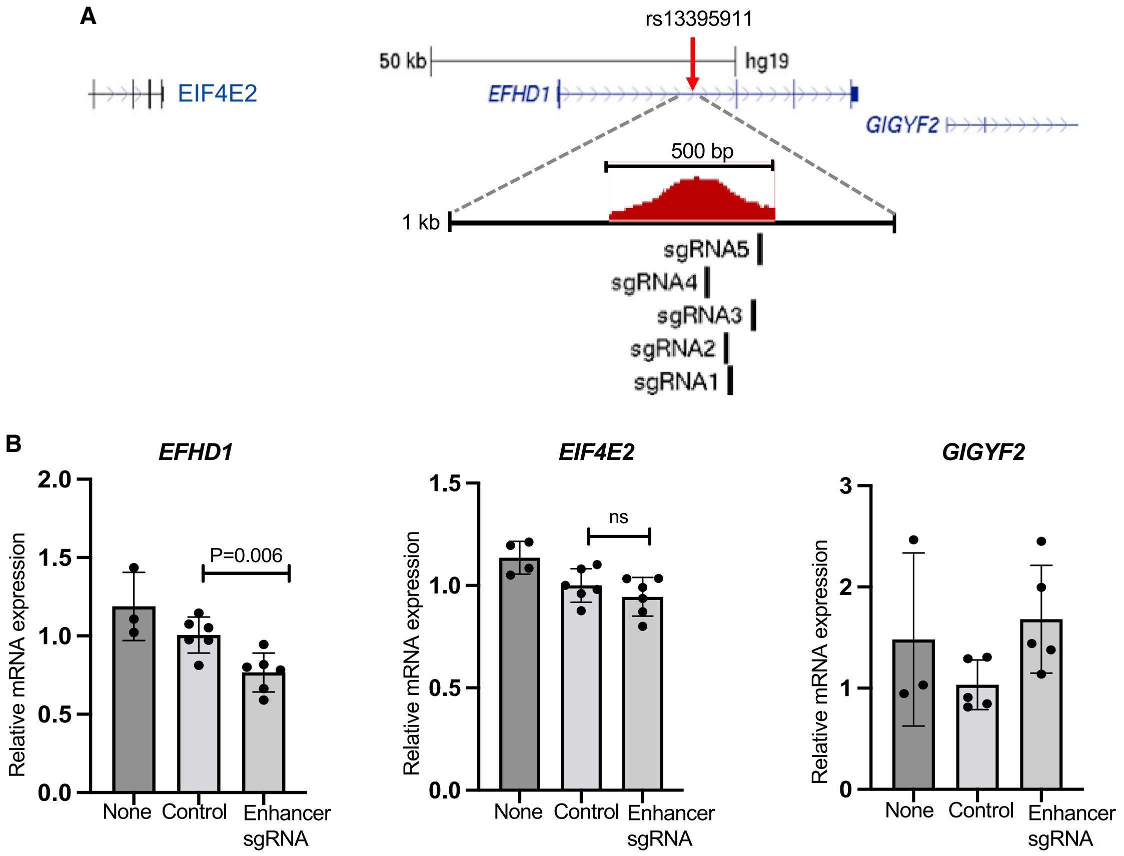

Figure 3. CRISPRi of the region spanning rs13395911 reduces *EFHD1* expression
(A) Mechanistic representation of CRISPRi for caSNP rs13395911 (red arrow), which is located between *EFHD1* exon 1 and exon 2 and overlaps the caQTL accessible chromatin. The enlarged region shows 5 sgRNAs designed to span the enhancer regulatory region.
(B) Gene expression measured after CRISPRi of the rs13395911 region. Compared to a pool of NTC (Control) sgRNAs, a pool of sgRNAs targeted to the enhancer led to lower expression of *EFHD1*, but not nearby genes *EIF4E2* and *GIGYF2*. "None" indicates cells with no sgRNAs. Each dot represents the mean of 3 qPCR replicates. Bars show the mean and SD of 4–6 biological replicates from different wells. p values are from 2-tailed t tests.

(Figure 3). At *LITAF*, compared to control cells transduced with NTC sgRNAs, cells with sgRNAs targeting the putative enhancer spanning rs11644920 showed 25% lower *LITAF* expression ($p = 0.006$) (Figure 4). At this signal, targeting the enhancer also reduced the expression of nearby genes *SNN* and *TXNDC11* by 39% ($p = 0.002$) and 26% ($p = 0.005$), respectively (Figure 4). These results suggest that the enhancer element spanning rs13395911 may be specific to *EFHD1*, whereas the enhancer spanning rs11644920 directly or indirectly affects *LITAF* as well as additional nearby genes. The CRISPRi experiments provided functional support of the regulatory element links to the *EFHD1* and *LITAF* genes and detected additional target genes at the *LITAF* signal.

EFHD1 metabolic activity in hepatocytes

Because the enhancer element spanning rs13395911 appeared specific to *EFHD1* and rs13395911 alleles disrupted binding of a transcription factor known to play a role in liver development and function, we tested the metabolic activity of *EFHD1* in hepatocyte cell lines. *EFHD1* regulates several mitochondrial activities in immune cells[41]; however, its role in hepatocytes is unknown. When cultured in glucose media, hepatocytes rely primarily on glycolysis to generate ATP, whereas culturing cells in galactose media requires them to produce ATP via mitochondrial oxidative phosphorylation in the tricarboxylic acid cycle pathway.[52] We cultured HepG2 and SNU-761 hepatocytes in two media concentrations of glucose and galactose (5.5 and 11.1 mM) and observed that *EFHD1* expression was increased 2- to 3-fold in galactose media compared to glucose media (Figure 5), suggesting that *EFHD1* is induced during oxidative phosphorylation in hepatocytes. We further evaluated the role of *EFHD1* on hepatic glucose and lipid homeostasis by knocking down *EFHD1* using shRNA sequences. Using cells with ≥82% knockdown of *EFHD1*, we performed assays for ATP production, lipid accumulation, and glucose uptake; however, we observed no significant differences compared to control HepG2 cells (Figure S5). These results suggest that although *EFHD1* is a metabolically active gene in hepatocytes and has an active enhancer element in liver, it may not act directly on these aspects of glucose or lipid metabolism to influence plasma levels of liver enzymes.

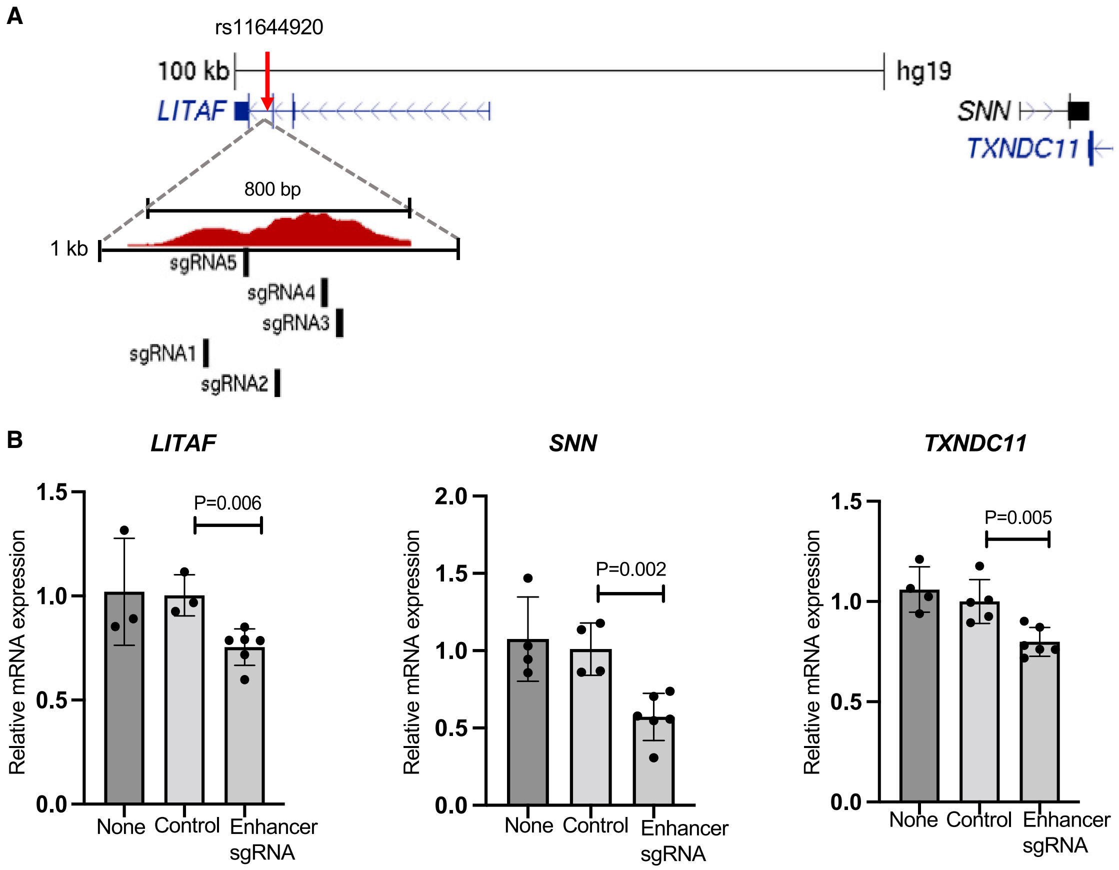

Figure 4. CRISPRi of the region spanning rs11644920 reduces expression of *LITAF*, *SNN*, and *TXNDC11*
(A) Mechanistic representation of CRISPRi for caSNP rs11644920. rs11644920 (red arrow) is located between exon 3 and exon 4 of *LITAF* and overlaps the caQTL accessible chromatin. The enlarged region shows 5 sgRNAs designed to span the enhancer regulatory region.
(B) Gene expression measured after CRISPRi of the rs11644920 region. Compared to a pool of NTC (Control) sgRNAs, a pool of sgRNAs targeted to the enhancer led to lower expression of *LITAF*, as well as nearby genes *SNN* and *TXNDC11*. None indicates cells with no sgRNAs. Each dot represents the mean of 3 qPCR replicates. Bars show the mean and SD of 4–6 biological replicates from different wells. p values are from 2-tailed t tests.

Discussion

To gain insight into the regulatory mechanisms of lipid and liver enzyme GWAS signals in noncoding regions, we studied five signals that had been colocalized with liver caQTL and eQTL.[11] The colocalizations predicted causal variants and target genes, but functional studies were needed to validate that variants exhibit differential allelic effects on gene expression and function and that distal regulatory elements affect predicted genes. We showed that caQTL variants exhibited significant allelic differences in transcriptional activity at signals located near *ZNF329*, *GPR180*, and *EFHD1*. The alleles that exhibited higher transcriptional activity for these signals were associated with greater chromatin accessibility in the liver caQTL study[11] and with a higher expression level of their corresponding target gene.[17] To identify target genes for two nonpromoter elements, we repressed the elements using CRISPRi and observed 20%–25% reduced expression of *EFHD1* and *LITAF*, as well as nearby genes to *LITAF*, *SNN*, and *TXDC11*. These results validate and expand predicted molecular mechanisms at four GWAS signals.

We further evaluated molecular and biological mechanisms at *EFHD1*. The T allele of the caQTL lead variant rs13395911 was associated with increased liver enzymes ALT, AST, and GGT.[4] We observed that rs13395911 displayed enhancer activity in HepG2 cells and that the rs13395911-T allele exhibited 3- to 4-fold higher transcriptional activity than the rs13395911-A allele. We further observed that the rs13395911-T allele bound more strongly to the transcription factor FOXA2, a nuclear protein that helps regulate liver lipid levels, because FOXA2 overexpression in mice has been shown to improve hepatic insulin sensitivity.[53] EFHD1 is a Ca^{2+}-binding HNF4A target protein known to be involved in early neuronal differentiation, axonal morphogenesis, and B cell activation.[41,42,54] Binding of FOXA2 to rs13395911 at this signal suggests that EFHD1 is a FOXA2 target protein and may influence glucose and lipid metabolism. Next, using CRISPRi, we showed that the enhancer element spanning rs13395911 appeared specific to *EFHD1* and did not affect the expression of two other adjacent genes. Finally, we showed that although *EFHD1* is a metabolically active gene in HepG2 cells and that its expression was induced by galactose, knockdown of *EFHD1* did not induce

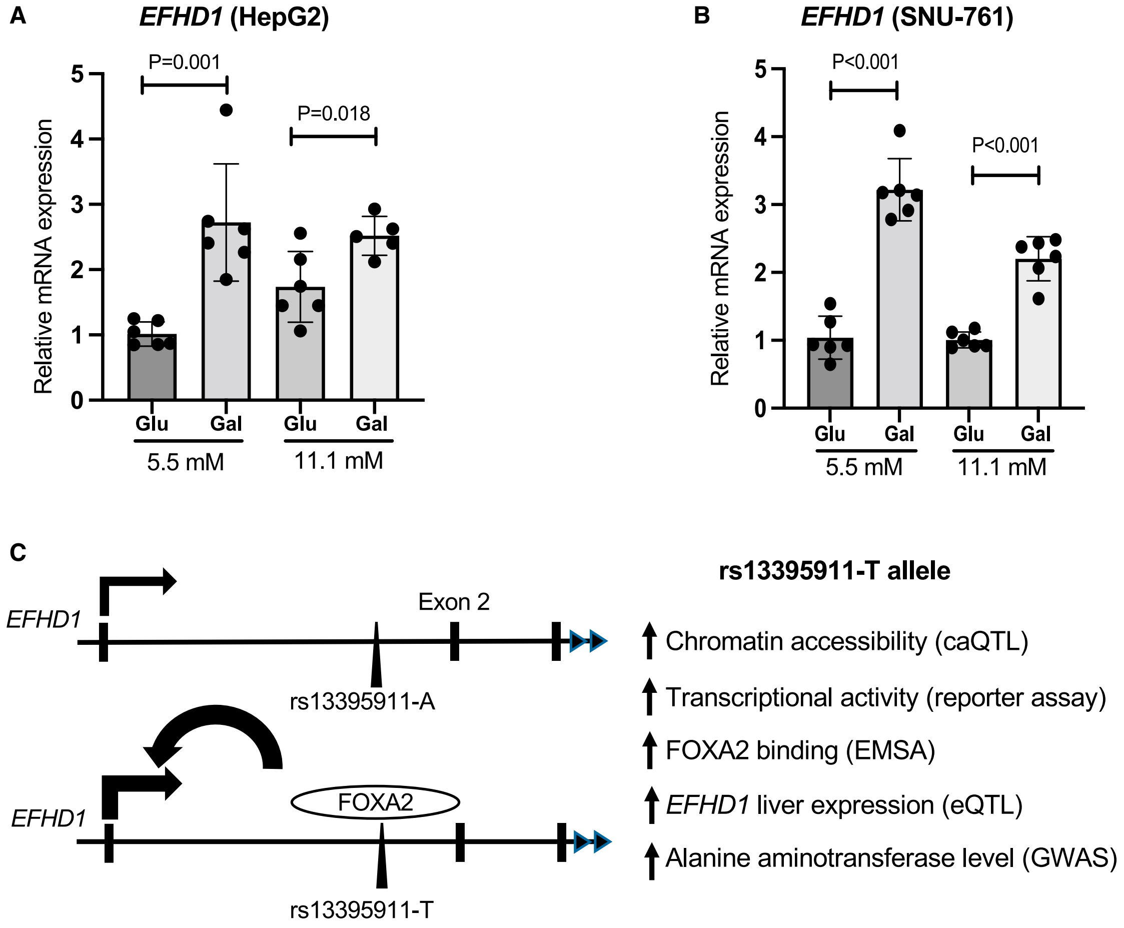

Figure 5. *EFHD1* molecular mechanism summary and metabolic induction by galactose
(A and B). After induction for 6–7 days in media containing galactose instead of glucose, *EFHD1* expression increased, especially at 11.1 mM galactose vs. 11.1 glucose in (A) HepG2 and (B) SNU761 cells. Each dot represents the mean of 3 qPCR triplicates, and bars show the mean and SD of 3–6 biological replicates in different wells. p values are from 2-tailed t tests.
(C) A proposed model for binding of FOXA2 to rs13395911-T leading to the upregulation of *EFHD1* expression and higher alanine aminotransferase levels.

direct effects on glucose uptake, ATP levels, or lipid accumulation. These results suggest that although *EFHD1* has an active enhancer element acting in liver, it may not act directly through the tested processes to alter plasma levels of liver enzymes.

Our analyses and experimental approaches have limitations. Our investigation relied on an *in vitro* cell model, HepG2, which is a human hepatoma cell line that is highly aneuploid[55] and may not fully represent the biology and complexity of hepatocytes, the liver, or its interaction with other organs. Similarly, although we pinpointed potential regulatory variants and identified their effects on gene expression, other variants at these signals may also affect transcriptional activity. Furthermore, when targeting elements within introns, transcriptional activity may be reduced due to the steric hinderance of RNA polymerase II by the CRISPR machinery,[56] which complicates identifying the effects of the element itself on transcription. Finally, our CRISPRi assays used a mixed cell population expressing the dCas9-KRAB system, limiting the repression of promoters and suggesting that the elements may be stronger enhancers than is shown by CRISPRi experiments. Further analysis of CRISPR-mediated knockins of specific alleles would more robustly test the effect of variants on function.

Together, the mechanisms and framework presented in this study provide a scalable approach for the identification and follow-up of additional GWAS signals. Integration of GWAS, caQTL, and eQTL signals can identify potential liver regulatory variants and their target genes, enhancing our understanding of liver biology and providing potential avenues for therapeutic intervention.

Data and code availability

The published article includes all of the datasets generated or analyzed during this study.

Supplemental information

Supplemental information can be found online at https://doi.org/10.1016/j.xhgg.2024.100275.

Acknowledgments

We thank the ENCODE Consortium, the ENCODE ChIP-seq production laboratories, and the ENCODE Data Coordination Center for generating and processing the transcription factor ChIP-seq datasets used here. This study was supported by the American Heart Association postdoctoral fellowship 903990 and National Institutes of Health grants R01DK072193, UM1DK126185, R01DK122503, T32HL129982, and T32GM135128.

Author contributions

G.K.P., K.W.C., and K.L.M. designed the study. G.K.P., S.V., A.H.M., J.N., and J.C.M. performed the experiments and analyzed the data. G.K.P. and K.L.M. interpreted the results. G.K.P. drafted the manuscript. G.K.P., K.W.C., K.A.B., and K.L.M. edited the manuscript. All of the authors reviewed and approved the final manuscript.

Declaration of interests

The authors declare no competing interests.

Received: October 1, 2023
Accepted: January 23, 2024

References

1. Cai, J., Zhang, X.-J., Ji, Y.-X., Zhang, P., She, Z.-G., and Li, H. (2020). Nonalcoholic fatty liver disease pandemic fuels the upsurge in cardiovascular diseases. Circ. Res. *126*, 679–704. https://doi.org/10.1161/CIRCRESAHA.119.316337.
2. Pazoki, R., Vujkovic, M., Elliott, J., Evangelou, E., Gill, D., Ghanbari, M., van der Most, P.J., Pinto, R.C., Wielscher, M., Farlik, M., et al. (2021). Genetic analysis in European ancestry individuals identifies 517 loci associated with liver enzymes. Nat. Commun. *12*, 2579. https://doi.org/10.1038/s41467-021-22338-2.
3. Graham, S.E., Clarke, S.L., Wu, K.-H.H., Kanoni, S., Zajac, G.J.M., Ramdas, S., Surakka, I., Ntalla, I., Vedantam, S., Winkler, T.W., et al. (2021). The power of genetic diversity in genome-wide association studies of lipids. Nature *600*, 675–679. https://doi.org/10.1038/s41586-021-04064-3.
4. Chen, V.L., Du, X., Chen, Y., Kuppa, A., Handelman, S.K., Vohnoutka, R.B., Peyser, P.A., Palmer, N.D., Bielak, L.F., Halligan, B., and Speliotes, E.K. (2021). Genome-wide association study of serum liver enzymes implicates diverse metabolic and liver pathology. Nat. Commun. *12*, 816. https://doi.org/10.1038/s41467-020-20870-1.
5. Klarin, D., Damrauer, S.M., Cho, K., Sun, Y.V., Teslovich, T.M., Honerlaw, J., Gagnon, D.R., DuVall, S.L., Li, J., Peloso, G.M., et al. (2018). Genetics of blood lipids among ∼300,000 multi-ethnic participants of the Million Veteran Program. Nat. Genet. *50*, 1514–1523. https://doi.org/10.1038/s41588-018-0222-9.
6. Cano-Gamez, E., and Trynka, G. (2020). From GWAS to function: using functional genomics to identify the mechanisms underlying complex diseases. Front. Genet. *11*, 424. https://doi.org/10.3389/fgene.2020.00424.
7. Maurano, M.T., Humbert, R., Rynes, E., Thurman, R.E., Haugen, E., Wang, H., Reynolds, A.P., Sandstrom, R., Qu, H., Brody, J., et al. (2012). Systematic localization of common disease-associated variation in regulatory DNA. Science *337*, 1190–1195. https://doi.org/10.1126/science.1222794.
8. Alasoo, K., Rodrigues, J., Mukhopadhyay, S., Knights, A.J., Mann, A.L., Kundu, K., HIPSCI Consortium, Hale, C., Dougan, G., and Gaffney, D.J. (2018). Shared genetic effects on chromatin and gene expression indicate a role for enhancer priming in immune response. Nat. Genet. *50*, 424–431. https://doi.org/10.1038/s41588-018-0046-7.
9. Gate, R.E., Cheng, C.S., Aiden, A.P., Siba, A., Tabaka, M., Lituiev, D., Machol, I., Gordon, M.G., Subramaniam, M., Shamim, M., et al. (2018). Genetic determinants of co-accessible chromatin regions in activated T cells across humans. Nat. Genet. *50*, 1140–1150. https://doi.org/10.1038/s41588-018-0156-2.
10. Khetan, S., Kursawe, R., Youn, A., Lawlor, N., Jillette, A., Marquez, E.J., Ucar, D., and Stitzel, M.L. (2018). Type 2 Diabetes-Associated Genetic Variants Regulate Chromatin Accessibility in Human Islets. Diabetes *67*, 2466–2477. https://doi.org/10.2337/db18-0393.
11. Currin, K.W., Erdos, M.R., Narisu, N., Rai, V., Vadlamudi, S., Perrin, H.J., Idol, J.R., Yan, T., Albanus, R.D., Broadaway, K.A., et al. (2021). Genetic effects on liver chromatin accessibility identify disease regulatory variants. Am. J. Hum. Genet. *108*, 1169–1189. https://doi.org/10.1016/j.ajhg.2021.05.001.
12. Gilbert, L.A., Larson, M.H., Morsut, L., Liu, Z., Brar, G.A., Torres, S.E., Stern-Ginossar, N., Brandman, O., Whitehead, E.H., Doudna, J.A., et al. (2013). CRISPR-mediated modular RNA-guided regulation of transcription in eukaryotes. Cell *154*, 442–451. https://doi.org/10.1016/j.cell.2013.06.044.
13. Mandegar, M.A., Huebsch, N., Frolov, E.B., Shin, E., Truong, A., Olvera, M.P., Chan, A.H., Miyaoka, Y., Holmes, K., Spencer, C.I., et al. (2016). CRISPR Interference Efficiently Induces Specific and Reversible Gene Silencing in Human iPSCs. Cell Stem Cell *18*, 541–553. https://doi.org/10.1016/j.stem.2016.01.022.
14. Gasperini, M., Hill, A.J., McFaline-Figueroa, J.L., Martin, B., Kim, S., Zhang, M.D., Jackson, D., Leith, A., Schreiber, J., Noble, W.S., et al. (2019). A Genome-wide Framework for Mapping Gene Regulation via Cellular Genetic Screens. Cell *176*, 377–390.e19. https://doi.org/10.1016/j.cell.2018.11.029.
15. Morris, J.A., Caragine, C., Daniloski, Z., Domingo, J., Barry, T., Lu, L., Davis, K., Ziosi, M., Glinos, D.A., Hao, S., et al. (2023). Discovery of target genes and pathways at GWAS loci by pooled single-cell CRISPR screens. Science *380*, eadh7699. https://doi.org/10.1126/science.adh7699.
16. Ren, X., Wang, M., Li, B., Jamieson, K., Zheng, L., Jones, I.R., Li, B., Takagi, M.A., Lee, J., Maliskova, L., et al. (2021). Parallel characterization of cis-regulatory elements for multiple genes using CRISPRpath. Sci. Adv. *7*, eabi4360. https://doi.org/10.1126/sciadv.abi4360.
17. Etheridge, A.S., Gallins, P.J., Jima, D., Broadaway, K.A., Ratain, M.J., Schuetz, E., Schadt, E., Schroder, A., Molony, C., Zhou, Y., et al. (2020). A New Liver Expression Quantitative Trait Locus Map From 1,183 Individuals Provides Evidence for Novel Expression Quantitative Trait Loci of Drug Response, Metabolic, and Sex-Biased Phenotypes. Clin. Pharmacol. Ther. *107*, 1383–1393. https://doi.org/10.1002/cpt.1751.
18. Kanai, M., Akiyama, M., Takahashi, A., Matoba, N., Momozawa, Y., Ikeda, M., Iwata, N., Ikegawa, S., Hirata, M., Matsuda, K., et al. (2018). Genetic analysis of quantitative traits in the Japanese population links cell types to complex human diseases. Nat. Genet. *50*, 390–400. https://doi.org/10.1038/s41588-018-0047-6.
19. Hoffmann, T.J., Theusch, E., Haldar, T., Ranatunga, D.K., Jorgenson, E., Medina, M.W., Kvale, M.N., Kwok, P.-Y., Schaefer,

C., Krauss, R.M., et al. (2018). A large electronic-health-record-based genome-wide study of serum lipids. Nat. Genet. *50*, 401–413. https://doi.org/10.1038/s41588-018-0064-5.

20. Jung, I., Schmitt, A., Diao, Y., Lee, A.J., Liu, T., Yang, D., Tan, C., Eom, J., Chan, M., Chee, S., et al. (2019). A compendium of promoter-centered long-range chromatin interactions in the human genome. Nat. Genet. *51*, 1442–1449. https://doi.org/10.1038/s41588-019-0494-8.
21. ENCODE Project Consortium (2012). An integrated encyclopedia of DNA elements in the human genome. Nature *489*, 57–74. https://doi.org/10.1038/nature11247.
22. Ernst, J., Kheradpour, P., Mikkelsen, T.S., Shoresh, N., Ward, L.D., Epstein, C.B., Zhang, X., Wang, L., Issner, R., Coyne, M., et al. (2011). Mapping and analysis of chromatin state dynamics in nine human cell types. Nature *473*, 43–49. https://doi.org/10.1038/nature09906.
23. ENCODE Project Consortium, Moore, J.E., Purcaro, M.J., Pratt, H.E., Epstein, C.B., Shoresh, N., Adrian, J., Kawli, T., Davis, C.A., Dobin, A., et al. (2020). Expanded encyclopaedias of DNA elements in the human and mouse genomes. Nature *583*, 699–710. https://doi.org/10.1038/s41586-020-2493-4.
24. Machiela, M.J., and Chanock, S.J. (2015). LDlink: a web-based application for exploring population-specific haplotype structure and linking correlated alleles of possible functional variants. Bioinformatics *31*, 3555–3557. https://doi.org/10.1093/bioinformatics/btv402.
25. Fogarty, M.P., Cannon, M.E., Vadlamudi, S., Gaulton, K.J., and Mohlke, K.L. (2014). Identification of a regulatory variant that binds FOXA1 and FOXA2 at the CDC123/CAMK1D type 2 diabetes GWAS locus. PLoS Genet. *10*, e1004633. https://doi.org/10.1371/journal.pgen.1004633.
26. Roman, T.S., Marvelle, A.F., Fogarty, M.P., Vadlamudi, S., Gonzalez, A.J., Buchkovich, M.L., Huyghe, J.R., Fuchsberger, C., Jackson, A.U., Wu, Y., et al. (2015). Multiple Hepatic Regulatory Variants at the GALNT2 GWAS Locus Associated with High-Density Lipoprotein Cholesterol. Am. J. Hum. Genet. *97*, 801–815. https://doi.org/10.1016/j.ajhg.2015.10.016.
27. Hazelbaker, D.Z., Beccard, A., Angelini, G., Mazzucato, P., Messana, A., Lam, D., Eggan, K., and Barrett, L.E. (2020). A multiplexed gRNA piggyBac transposon system facilitates efficient induction of CRISPRi and CRISPRa in human pluripotent stem cells. Sci. Rep. *10*, 635. https://doi.org/10.1038/s41598-020-57500-1.
28. Smith, G.A., Padmanabhan, A., Lau, B.H., Pampana, A., Li, L., Lee, C.Y., Pelonero, A., Nishino, T., Sadagopan, N., Xia, V.Q., et al. (2022). Cold shock domain-containing protein E1 is a posttranscriptional regulator of the LDL receptor. Sci. Transl. Med. *14*, eabj8670.
29. Ran, F.A., Hsu, P.D., Wright, J., Agarwala, V., Scott, D.A., and Zhang, F. (2013). Genome engineering using the CRISPR-Cas9 system. Nat. Protoc. *8*, 2281–2308. https://doi.org/10.1038/nprot.2013.143.
30. Elegheert, J., Behiels, E., Bishop, B., Scott, S., Woolley, R.E., Griffiths, S.C., Byrne, E.F.X., Chang, V.T., Stuart, D.I., Jones, E.Y., et al. (2018). Lentiviral transduction of mammalian cells for fast, scalable and high-level production of soluble and membrane proteins. Nat. Protoc. *13*, 2991–3017. https://doi.org/10.1038/s41596-018-0075-9.
31. Moore, C.B., Guthrie, E.H., Huang, M.T.-H., and Taxman, D.J. (2010). Short hairpin RNA (shRNA): design, delivery, and assessment of gene knockdown. Methods Mol. Biol. *629*, 141–158. https://doi.org/10.1007/978-1-60761-657-3_10.
32. Perrin, H.J., Currin, K.W., Vadlamudi, S., Pandey, G.K., Ng, K.K., Wabitsch, M., Laakso, M., Love, M.I., and Mohlke, K.L. (2021). Chromatin accessibility and gene expression during adipocyte differentiation identify context-dependent effects at cardiometabolic GWAS loci. PLoS Genet. *17*, e1009865. https://doi.org/10.1371/journal.pgen.1009865.
33. Lee, J.S., Hmama, Z., Mui, A., and Reiner, N.E. (2004). Stable gene silencing in human monocytic cell lines using lentiviral-delivered small interference RNA. Silencing of the p110alpha isoform of phosphoinositide 3-kinase reveals differential regulation of adherence induced by 1alpha,25-dihydroxycholecalciferol and bacterial lipopolysaccharide. J. Biol. Chem. *279*, 9379–9388. https://doi.org/10.1074/jbc.M310638200.
34. Orlicka-Płocka, M., Gurda-Wozna, D., Fedoruk-Wyszomirska, A., and Wyszko, E. (2020). Circumventing the Crabtree effect: forcing oxidative phosphorylation (OXPHOS) via galactose medium increases sensitivity of HepG2 cells to the purine derivative kinetin riboside. Apoptosis. *25*, 835–852. https://doi.org/10.1007/s10495-020-01637-x.
35. Li, P., Liu, S., Lu, M., Bandyopadhyay, G., Oh, D., Imamura, T., Johnson, A.M.F., Sears, D., Shen, Z., Cui, B., et al. (2016). Hematopoietic-Derived Galectin-3 Causes Cellular and Systemic Insulin Resistance. Cell *167*, 973–984.e12. https://doi.org/10.1016/j.cell.2016.10.025.
36. Arredouani, A., Diane, A., Khattab, N., Bensmail, I., Aoude, I., Chikri, M., Mohammad, R., Abou-Samra, A.B., and Dehbi, M. (2019). DNAJB3 attenuates metabolic stress and promotes glucose uptake by eliciting Glut4 translocation. Sci. Rep. *9*, 4772. https://doi.org/10.1038/s41598-019-41244-8.
37. Pandey, G.K., Vadivel, S., Raghavan, S., Mohan, V., Balasubramanyam, M., and Gokulakrishnan, K. (2019). High molecular weight adiponectin reduces glucolipotoxicity-induced inflammation and improves lipid metabolism and insulin sensitivity via APPL1-AMPK-GLUT4 regulation in 3T3-L1 adipocytes. Atherosclerosis *288*, 67–75. https://doi.org/10.1016/j.atherosclerosis.2019.07.011.
38. Beacon, T.H., Delcuve, G.P., López, C., Nardocci, G., Kovalchuk, I., van Wijnen, A.J., and Davie, J.R. (2021). The dynamic broad epigenetic (H3K4me3, H3K27ac) domain as a mark of essential genes. Clin. Epigenetics *13*, 138. https://doi.org/10.1186/s13148-021-01126-1.
39. Luo, Y., Hitz, B.C., Gabdank, I., Hilton, J.A., Kagda, M.S., Lam, B., Myers, Z., Sud, P., Jou, J., Lin, K., et al. (2020). New developments on the Encyclopedia of DNA Elements (ENCODE) data portal. Nucleic Acids Res. *48*, D882–D889. https://doi.org/10.1093/nar/gkz1062.
40. Woo, Y.H., and Li, W.-H. (2012). Evolutionary conservation of histone modifications in mammals. Mol. Biol. Evol. *29*, 1757–1767. https://doi.org/10.1093/molbev/mss022.
41. Stein, M., Dütting, S., Mougiakakos, D., Bösl, M., Fritsch, K., Reimer, D., Urbanczyk, S., Steinmetz, T., Schuh, W., Bozec, A., et al. (2017). A defined metabolic state in pre B cells governs B-cell development and is counterbalanced by Swiprosin-2/EFhd1. Cell Death Differ. *24*, 1239–1252. https://doi.org/10.1038/cdd.2017.52.
42. Ulisse, V., Dey, S., Rothbard, D.E., Zeevi, E., Gokhman, I., Dadosh, T., Minis, A., and Yaron, A. (2020). Regulation of axonal morphogenesis by the mitochondrial protein

Efhd1. Life Sci. Alliance *3*, e202000753. https://doi.org/10.26508/lsa.202000753.
43. Balazova, L., Balaz, M., Horvath, C., Horváth, Á., Moser, C., Kovanicova, Z., Ghosh, A., Ghoshdastider, U., Efthymiou, V., Kiehlmann, E., et al. (2021). GPR180 is a component of TGFβ signalling that promotes thermogenic adipocyte function and mediates the metabolic effects of the adipocyte-secreted factor CTHRC1. Nat. Commun. *12*, 7144. https://doi.org/10.1038/s41467-021-27442-x.
44. Chambers, J.C., Zhang, W., Sehmi, J., Li, X., Wass, M.N., Van der Harst, P., Holm, H., Sanna, S., Kavousi, M., Baumeister, S.E., et al. (2011). Genome-wide association study identifies loci influencing concentrations of liver enzymes in plasma. Nat. Genet. *43*, 1131–1138. https://doi.org/10.1038/ng.970.
45. Gancheva, S., Ouni, M., Jelenik, T., Koliaki, C., Szendroedi, J., Toledo, F.G.S., Markgraf, D.F., Pesta, D.H., Mastrototaro, L., De Filippo, E., et al. (2019). Dynamic changes of muscle insulin sensitivity after metabolic surgery. Nat. Commun. *10*, 4179. https://doi.org/10.1038/s41467-019-12081-0.
46. Tsukada, S., Iwai, M., Nishiu, J., Itoh, M., Tomoike, H., Horiuchi, M., Nakamura, Y., and Tanaka, T. (2003). Inhibition of experimental intimal thickening in mice lacking a novel G-protein-coupled receptor. Circulation *107*, 313–319. https://doi.org/10.1161/01.cir.0000043804.29963.b4.
47. Bertolo, C., Roa, S., Sagardoy, A., Mena-Varas, M., Robles, E.F., Martinez-Ferrandis, J.I., Sagaert, X., Tousseyn, T., Orta, A., Lossos, I.S., et al. (2013). LITAF, a BCL6 target gene, regulates autophagy in mature B-cell lymphomas. Br. J. Haematol. *162*, 621–630. https://doi.org/10.1111/bjh.12440.
48. Husain, A., Chiu, Y.-T., Sze, K.M.-F., Ho, D.W.-H., Tsui, Y.-M., Suarez, E.M.S., Zhang, V.X., Chan, L.-K., Lee, E., Lee, J.M.-F., et al. (2022). Ephrin-A3/EphA2 axis regulates cellular metabolic plasticity to enhance cancer stemness in hypoxic hepatocellular carcinoma. J. Hepatol. *77*, 383–396. https://doi.org/10.1016/j.jhep.2022.02.018.
49. Park, J.E., Son, A.I., and Zhou, R. (2013). Roles of epha2 in development and disease. Genes *4*, 334–357. https://doi.org/10.3390/genes4030334.
50. Gerstein, M.B., Kundaje, A., Hariharan, M., Landt, S.G., Yan, K.-K., Cheng, C., Mu, X.J., Khurana, E., Rozowsky, J., Alexander, R., et al. (2012). Architecture of the human regulatory network derived from ENCODE data. Nature *489*, 91–100. https://doi.org/10.1038/nature11245.
51. Aghadi, M., Elgendy, R., and Abdelalim, E.M. (2022). Loss of FOXA2 induces ER stress and hepatic steatosis and alters developmental gene expression in human iPSC-derived hepatocytes. Cell Death Dis. *13*, 713. https://doi.org/10.1038/s41419-022-05158-0.
52. Marroquin, L.D., Hynes, J., Dykens, J.A., Jamieson, J.D., and Will, Y. (2007). Circumventing the Crabtree effect: replacing media glucose with galactose increases susceptibility of HepG2 cells to mitochondrial toxicants. Toxicol. Sci. *97*, 539–547. https://doi.org/10.1093/toxsci/kfm052.
53. Zhang, L., Rubins, N.E., Ahima, R.S., Greenbaum, L.E., and Kaestner, K.H. (2005). Foxa2 integrates the transcriptional response of the hepatocyte to fasting. Cell Metab *2*, 141–148. https://doi.org/10.1016/j.cmet.2005.07.002.
54. Dütting, S., Brachs, S., and Mielenz, D. (2011). Fraternal twins: Swiprosin-1/EFhd2 and Swiprosin-2/EFhd1, two homologous EF-hand containing calcium binding adaptor proteins with distinct functions. Cell Commun. Signal. *9*, 2. https://doi.org/10.1186/1478-811X-9-2.
55. Arzumanian, V.A., Kiseleva, O.I., and Poverennaya, E.V. (2021). The curious case of the hepg2 cell line: 40 years of expertise. Int. J. Mol. Sci. *22*, 13135. https://doi.org/10.3390/ijms222313135.
56. Larson, M.H., Gilbert, L.A., Wang, X., Lim, W.A., Weissman, J.S., and Qi, L.S. (2013). CRISPR interference (CRISPRi) for sequence-specific control of gene expression. Nat. Protoc. *8*, 2180–2196. https://doi.org/10.1038/nprot.2013.132.

HGGA, Volume *5*

Supplemental information

Liver regulatory mechanisms of noncoding variants at lipid and metabolic trait loci

Gautam K. Pandey, Swarooparani Vadlamudi, Kevin W. Currin, Anne H. Moxley, Jayna C. Nicholas, Jessica C. McAfee, K. Alaine Broadaway, and Karen L. Mohlke

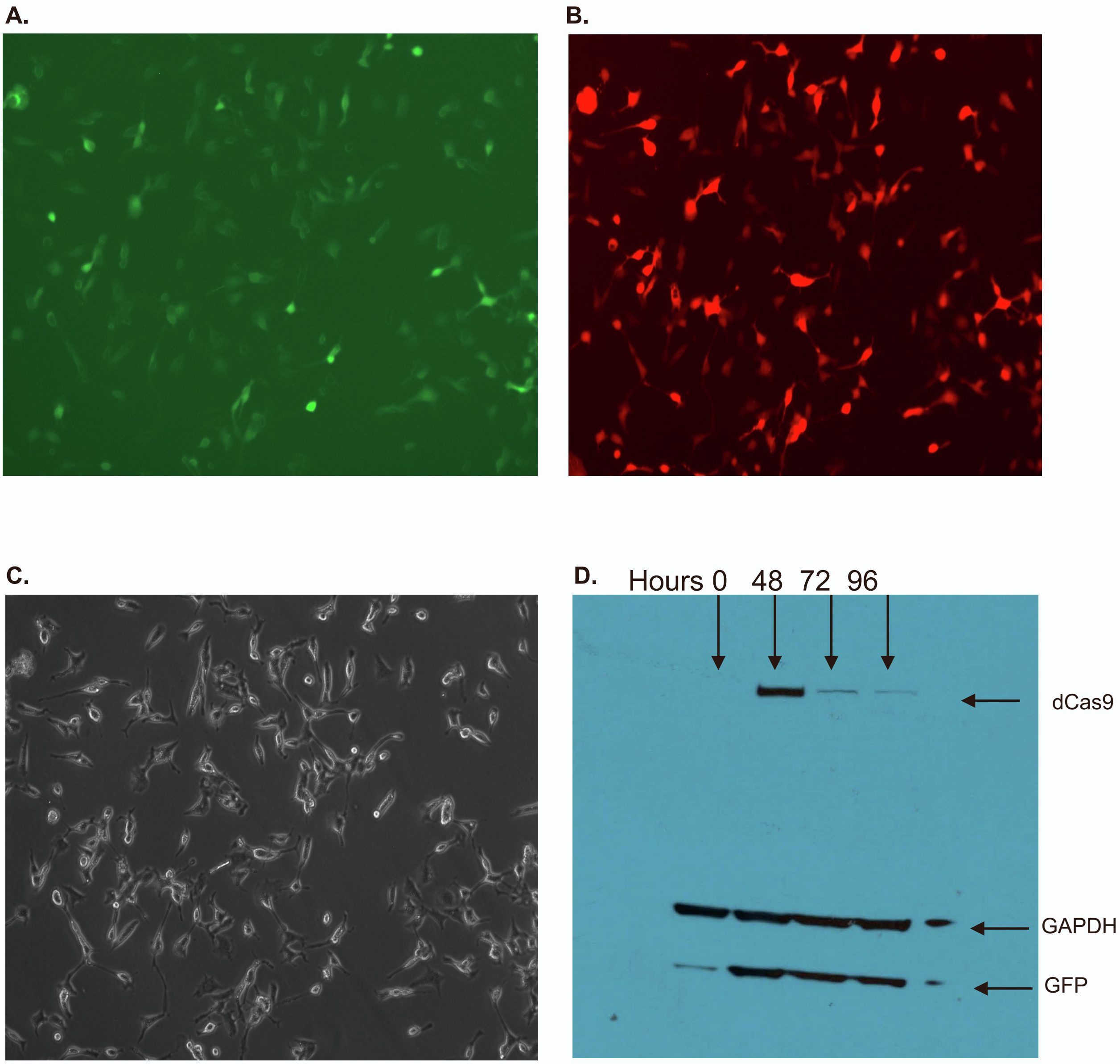

Figure S1. HepG2 cells expressing dCas9-KRAB and GFP.
(A) Expression of GFP 40 hours after activation of dCas9-KRAB and GFP by doxycycline. (B) Expression of mCherry 48 hours after infection with lentiviruses expressing sgRNAs and mCherry. (C) Bright light image of same focal area. (D) dCas9 protein levels at 48, 72 and 96 hours after activation by doxycycline. The highest expression of dCas9 protein was observed 48 hours after activation.

A.

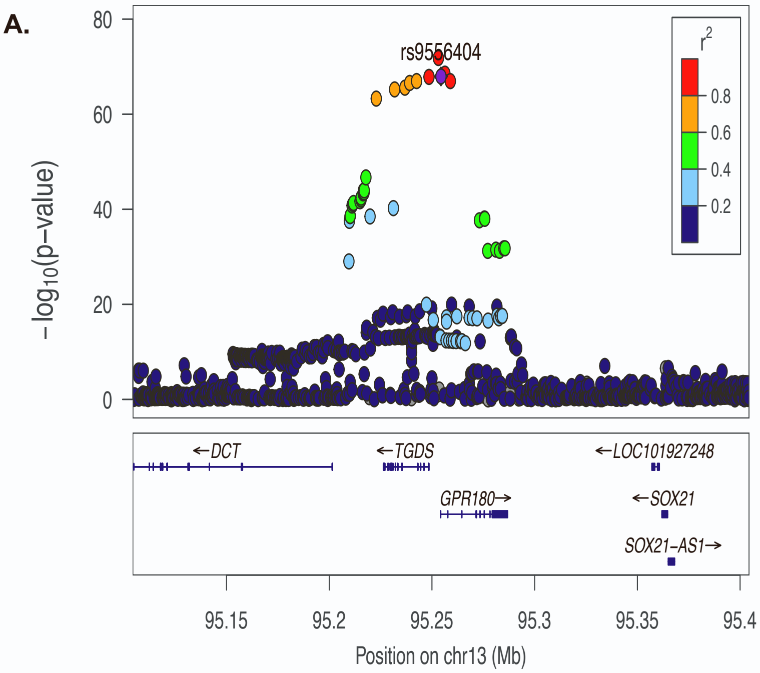

B.

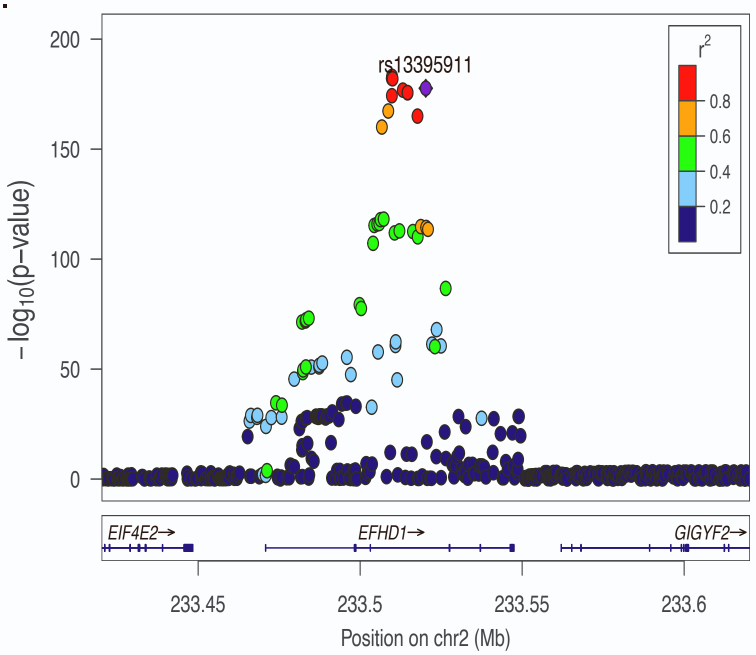

Figure S2. Liver eQTL plots for *GPR180* and *EFHD1*. Plots are colored based on LD with caQTL variants (A) rs9556404 and (B) rs13395911. Data are from Etheridge *et al.,* 2020.

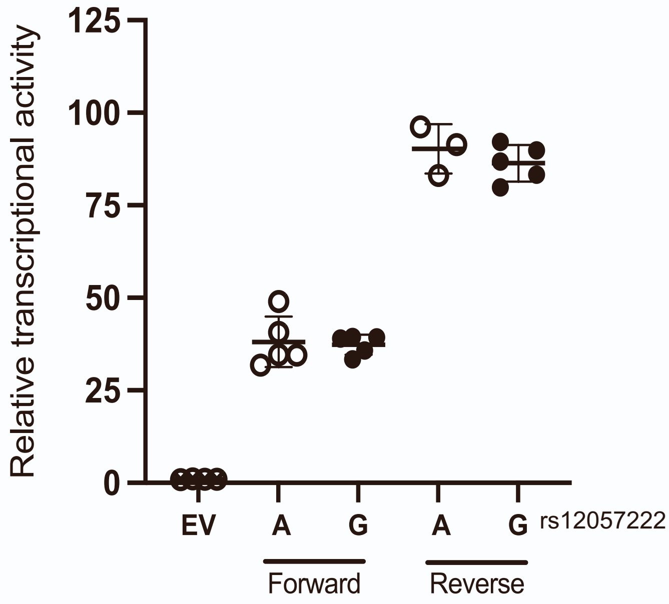

Figure S3. Transcriptional reporter activity for caQTL variant rs12057222 near *EPHA2*. A DNA element spanning rs12057222-A or rs12057222-G showed ~25 to 90-fold enhancer activity compared to empty vector (EV) control, but no allelic differences in transcriptional activity in HepG2 cells. Dots in the scatter plot represent 4-5 independent clones per allele with mean and standard deviation.

A.

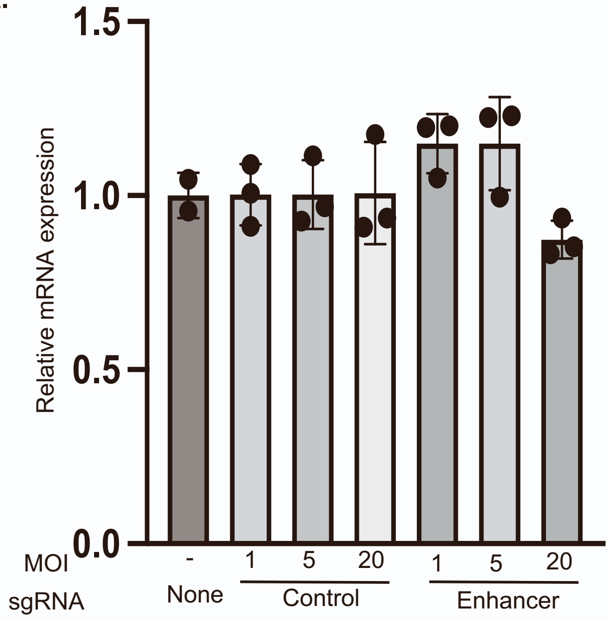

B.

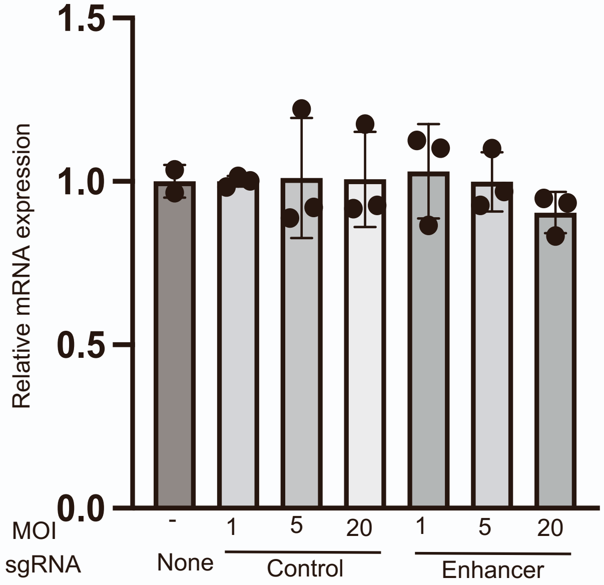

Figure S4. CRISPRi of enhancers shows less reduction of gene expression after 7 days. (A) Expression of *EFHD1* 7 days after CRISPRi of the region spanning rs13395911 at MOI of 1, 5, and 20. (B) Expression of *LITAF* 7 days after CRISPRi of the region spanning rs11644920 at MOI of 1, 5, and 20. qPCR was performed 7 days after transduction. Compared to pools of non-targeted control (Control) sgRNAs, pools of sgRNAs targeted to the enhancers did not significantly reduce gene expression. None indicates cells with no sgRNAs. Each dot represents the mean of three qPCR replicates. Bars show the mean and standard deviation of 3 biological replicates from different wells.

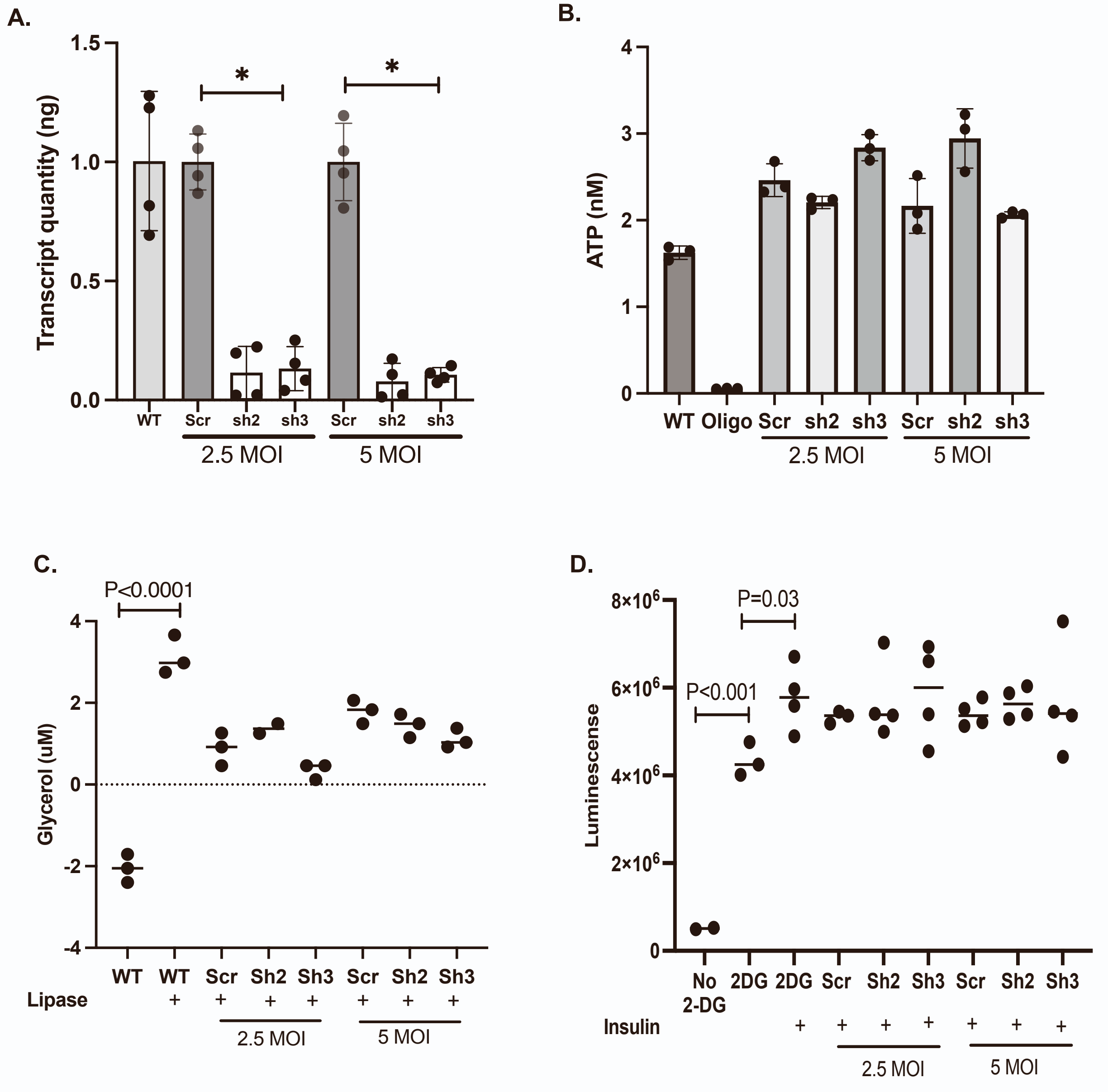

Figure S5. Effect of *EFHD1* knockdown on metabolic assays. (A) Confirmation of *EFHD1* knockdown in HepG2 cells by qPCR of *EFHD1* at 2 multiplicities of infection (MOI) with 2 shRNA constructs targeting different exons (sh2, sh3) compared to their respective scrambled controls (Scr). *P <0.05. (B) No effect of *EFHD1* knockdown on cellular ATP production. Bars represent mean and standard deviation of 3-4 biological replicates. (C) No effect on lipid accumulation after 96 hours of *EFHD1* knockdown. (D) No effect on glucose uptake after 96 hours of *EFHD1* knockdown. Scatter plot represents mean and standard deviation of 3-4 biological replicates. Each dot represents a single transduction event or biological replicate. p-values from 2-tailed t-tests.

Table S1. Characteristics of liver caQTL signals selected for study

caQTL variants studied	Lead caQTL variant	Location of caQTL variants studied	GWAS variant	Number of GWAS proxies ($r^2 \geq .8$)	GWAS p-value	Effect allele	Direction of effect on GWAS trait	References for effect on GWAS trait	eQTL gene	Direction of effect on target gene expression based on Etheridge et al 2020.	Target gene prediction
rs13395911	rs13395911	*EFHD1* intron	rs13395911	11	2E-16	T	Higher AST & ALT levels	Chen 2021, Kanai 2018	*EFHD1*	Higher *EFHD1*	eQTL, Hi-C
rs11644920	rs57792815	*LITAF* intron	rs34318965	40	3E-10	T	Higher LDL-C levels	Klarin 2018	*LITAF*	Lower *LITAF*	eQTL, Hi-C
rs34003091 & rs35081008	rs34003091	~105 bp upstream of *ZNF329*	rs34503352	6	3E-20	rs35081008-C	Higher LDL-C levels	Richardson 2020	*ZNF329*	Higher *ZNF329*	eQTL
rs9556404	rs6492720	~200 bp downstream of *GPR180* promoter	rs2298058	10	8E-09	A	Higher triglyceride levels	Hoffmann 2018	*GPR180*	Higher *GPR180*	eQTL
rs12057222	rs36086195	*EPHA2* intron	rs1497406	32	3.00E-19	G	Higher ALT levels	Chen 2021	*EPHA2*	Lower *EPHA2*	eQTL

caQTL variants studied are located within the caQTL peak, except rs12057222 is located in the adjacent peak. At ZNF329, variants rs34003091 and rs35081008 are 20 bp apart. HiC refers to promoter capture HiC (Jung et al 2019), eQTL data are from Etheridge et al., 2020. GWAS references were used to determine the direction of effect of the variant association with the GWAS traits. LDL-C, low density lipoprotein cholesterol; ALT, alanine aminotransferase; GGT, gamma glutamyltransferase; AST, aspartate aminotransferase.

Table S2. Primer sequences used in transcriptional reporter and electrophoretic mobility shift assays

Primer sequences for transcriptional reporter assays

eQTL Gene	PCR primer	Sequence (5’ -- 3’)	Amplified regions (hg19)
EPHA2	rs12057222_Forward<br>rs12057222_Reverse	GCGCGGTACCGGTCCTGAGTTCCCTTATCCC<br>GCGCGCTAGCCTCCCATGACCTTCCCATCC	chr1:16508335+16508728
EFHD1	rs13395911_Forward<br>rs13395911_Reverse	GCGCGGTACCGCATTGCCAAGGTACGTCTT<br>GCGCCTCGAGGCCTTCAGATGTTGCCCAG	chr2:233520112+233520454
GPR180	rs9556404_Forward<br>rs9556404_Reverse	GCGCGGTACCAGGGCAGCCAGGGTAAGAC<br>GCGCCTCGAGCACGATCACTAGTGGCGAGA	chr13:95254234+95254508
ZNF329	2SNPHap_Fwd<br>2SNPHap_Reverse	GCGCAAGCTTTCCACCCGCCAAGACTTC<br>GCGCCTCGAGTCGGGTTAAAAATCAGAGGAGA	chr19:58662049+58662332

Primers are shown 5'-3' with respect to the genome. The 2SNPHap includes rs34003091 and rs35081008

Oligonucleotide sequences for electrophoretic mobility shift assays

EFHD1	rs13395911_Forward	AACCTGTT[A/T]ACTTTGAC	chr2:233520246-233520262

Table S3. Primer sequences used to clone gRNAs

#Guide ID	Target Sequence	MIT Sperc Score	CFD Spec Score	Off Target Count	Final sequence after adding BbSI restriction site (5'--3')
EFHD1_176_Forward<br>EFHD1_176_Reverse	TGGACACATACGGAAGGCTC *TGG*	90	94	68	CACCGTGGACACATACGGAAGGCTC<br>AAACGAGCCTTCCGTATGTGTCCAC
EFHD1_141_Forward<br>EFHD1_141_Reverse	TGTGTCCAAAACTTACAAGA *GGG*	76	87	162	CACCGTGTGTCCAAAACTTACAAG<br>AAACTCTTGTAAGTTTTGGACACAC
EFHD1_240_Forward<br>EFHD1_240_Reverse	GAGAGCTTGCACAAACCGGC *TGG*	94	96	51	CACCGAGAGCTTGCACAAACCGGC<br>AAACGCCGGTTTGTGCAAGCTCTC
EFHD1_65_Forward<br>EFHD1_65_Reverse	TCTGTTGAGAAGACGTACCT *TGG*	93	94	76	CACCGTCTGTTGAGAAGACGTACCT<br>AAACAGGTACGTCTTCTCAACAGAC
EFHD1_253_Forward<br>EFHD1_253_Reverse	TTCTCAGGTCTAGTTCCAGT *TGG*	73	83	259	CACCGTTCTCAGGTCTAGTTCCAGT<br>AAACACTGGAACTAGACCTGAGAAC

#Guide ID	Target Sequence	MIT Sperc Score	CFD Spec Score	Off Target Count	Final sequence after adding BbSI restriction site (5'--3')
LITAF_128-Forward<br>LITAF_128-Reverse	ACTTTCATCCTGATGAGCCA *TGG*	73	86	150	CACCGACTTTCATCCTGATGAGCCA<br>AAACTGGCTCATCAGGATGAAAGTC
LITAF_374-Forward<br>LITAF_374-Reverse	AATCCACCTTTGGCACACAC *AGG*	75	91	122	CACCGAATCCACCTTTGGCACACAC<br>AAACGTGTGTGCCAAAGGTGGATTC
LITAF_585-Forward<br>LITAF_585-Reverse	TCAGCAGGAATCCTAACCTA *TGG*	82	91	91	CACCGTCAGCAGGAATCCTAACCTA<br>AAACTAGGTTAGGATTCCTGCTGAC
LITAF_259-Forward<br>LITAF_259-Reverse	GAGATCAGTAAGACGGGGGT *TGG*	84	88	139	CACCGAGATCAGTAAGACGGGGGT<br>AAACACCCCCGTCTTACTGATCTC
LITAF_315-Forward<br>LITAF_315-Reverse	ATTATTTGGGGAACTCTACT *TGG*	81	88	140	CACCGATTATTTGGGGAACTCTACT<br>AAACAGTAGAGTTCCCCAAATAATC
LITAF_515-Forward<br>LITAF_515-Reverse	TGGAAAGCTTAGGCAATAGG *AGG*	72	86	191	CACCGTGGAAAGCTTAGGCAATAGG<br>AAACCCTATTGCCTAAGCTTTCCAC
